# Cord Blood Proteomic Profiles, Birth Weight, and Early Life Growth Trajectories

**DOI:** 10.1001/jamanetworkopen.2024.11246

**Published:** 2024-05-14

**Authors:** Thessa Van Pee, Dries S. Martens, Rossella Alfano, Liesa Engelen, Hanne Sleurs, Leen Rasking, Michelle Plusquin, Tim S. Nawrot

**Affiliations:** 1 Centre for Environmental Sciences, Hasselt University, Diepenbeek, Belgium; 2 Department of Public Health and Primary Care, Leuven University, Leuven, Belgium

## Abstract

**Importance:**

The cord blood proteome, a repository of proteins derived from both mother and fetus, might offer valuable insights into the physiological and pathological state of the fetus. However, its association with birth weight and growth trajectories early in life remains unexplored.

**Objective:**

To identify cord blood proteins associated with birth weight and the birth weight ratio (BWR) and to evaluate the associations of these cord blood proteins with early growth trajectories.

**Design, Setting, and Participants:**

This cohort study included 288 mother-child pairs from the ongoing prospective Environmental Influence on Early Aging birth cohort study. Newborns were recruited from East-Limburg Hospital in Genk, Belgium, between February 2010 and November 2017 and followed up until ages 4 to 6 years. Data were analyzed from February 2022 to September 2023.

**Main Outcomes and Measures:**

The outcome of interest was the associations of 368 inflammatory-related cord blood proteins with birth weight or BWR and with early life growth trajectories (ie, rapid growth at age 12 months and weight, body mass index [BMI] *z *score, waist circumference, and overweight at age 4-6 years) using multiple linear regression models. The BWR was calculated by dividing the birth weight by the median birth weight of the population-specific reference growth curve, considering parity, sex, and gestational age. Results are presented as estimates or odds ratios (ORs) for each doubling in proteins.

**Results:**

The sample included 288 infants (125 [43.4%] male; mean [SD] gestation age, 277.2 [11.6] days). The mean (SD) age of the child at the follow-up examination was 4.6 (0.4) years old. After multiple testing correction, there were significant associations of birth weight and BWR with 7 proteins: 2 positive associations: afamin (birth weight: coefficient, 341.16 [95% CI, 192.76 to 489.50]) and secreted frizzled-related protein 4 (SFRP4; birth weight: coefficient, 242.60 [95% CI, 142.77 to 342.43]; BWR: coefficient, 0.07 [95% CI, 0.04 to 0.10]) and 5 negative associations: cadherin EGF LAG 7-pass G-type receptor 2 (CELSR2; birth weight: coefficient, −237.52 [95% CI, −343.15 to −131.89]), ephrin type-A receptor 4 (EPHA4; birth weight: coefficient, −342.78 [95% CI, −463.10 to −222.47]; BWR: coefficient, −0.11 [95% CI, −0.14 to −0.07]), SLIT and NTRK-like protein 1 (SLITRK1; birth weight: coefficient, −366.32 [95% CI, −476.66 to −255.97]; BWR: coefficient, −0.11 [95% CI, −0.15 to −0.08]), transcobalamin-1 (TCN1; birth weight: coefficient, −208.75 [95% CI, −305.23 to −112.26]), and unc-5 netrin receptor D (UNC5D; birth weight: coefficient, −209.27 [95% CI, −295.14 to −123.40]; BWR: coefficient, −0.07 [95% CI, −0.09 to −0.04]). Further evaluation showed that 2 proteins were still associated with rapid growth at age 12 months (afamin: OR, 0.32 [95% CI, 0.11-0.88]; TCN1: OR, 2.44 [95% CI, 1.26-4.80]). At age 4 to 6 years, CELSR2, EPHA4, SLITRK1, and UNC5D were negatively associated with weight (coefficients, −1.33 to −0.68 kg) and body mass index *z* score (coefficients, −0.41 to −0.23), and EPHA4, SLITRK1, and UNC5D were negatively associated with waist circumference (coefficients, −1.98 to −0.87 cm). At ages 4 to 6 years, afamin (OR, 0.19 [95% CI, 0.05-0.70]) and SLITRK1 (OR, 0.32 [95% CI, 0.10-0.99]) were associated with lower odds for overweight.

**Conclusions and Relevance:**

This cohort study found 7 cord blood proteins associated with birth weight and growth trajectories early in life. Overall, these findings suggest that stressors that could affect the cord blood proteome during pregnancy might have long-lasting associations with weight and body anthropometrics.

## Key Points

**Question:**

Are cord blood protein levels associated with birth weight and growth trajectories early in life?

**Findings:**

This cohort study including 288 longitudinally followed-up newborns found that 2 cord blood proteins were positively associated with birth weight and/or the birth weight ratio and 5 cord blood proteins were negatively associated. Most of these proteins were still associated with rapid growth at 12 months and weight, body mass index *z* score, waist circumference, and/or overweight at ages 4 to 6 years.

**Meaning:**

These findings suggest that cord blood proteins might be associated with birth weight and growth.

## Teaser

This cohort study examines cord blood proteins in association with birth weight and early growth trajectories among infants in Belgium.

## Introduction

Birth weight is associated with in utero health, as it mirrors, among other factors, the nutritional condition of the mother and development of the fetus.^1,2^ Factors such as the infant’s sex,^3^ maternal diet^4^ and smoking^5^ during pregnancy, pregnancy complications (eg, diabetes gravidarum, preeclampsia, or gestational hypertension),^6,7,8^ and socioeconomic status^9^ are associated with full-term birth weight.^10^ Approximately 9% of infants are born with a too-high birth weight (>4000 g)^11^ and 15% to 20% are born with too-low birth weight (<2500 g).^12^ Both deviations are public health concerns, as they are associated with short- and long-term health consequences, eg, neonatal mortality,^2,13^ childhood hypertension,^14^ and type 1 and 2 diabetes.^14^ Additionally, low and high birth weight are both associated with overweight^15,16^ and its associated diseases^17^ later in life, eg, high blood pressure and osteoarthritis, further supporting the developmental origins of health and disease hypothesis.^18^

Proteomics is increasingly used to detect physiological changes associated with growth,^19,20,21,22^ as a clear association with changes in the plasma proteome has been observed during all stages of development.^19,23,24^ For example, Suski et al^19^ studied the difference in plasma proteome between preterm newborns stratified by their gestational age and found significant differences in proteins belonging to the inflammatory, immunomodulatory, and coagulation domains. In addition, various cord blood proteins have been reported as associated with birth weight in single-protein studies.^25,26^ Nevertheless, the long-term outcomes associated with the cord blood proteome remain understudied. Cord blood contains proteins secreted from almost all tissues of both mother and fetus and is associated with the physiological and pathological state of the fetus.^27^ In this study, we present the first large cord blood proteomics analysis of birth weight and child growth trajectories, to our knowledge. We investigate the associations of 368 inflammatory-related cord blood proteins and infant birth weight and birth weight ratio (BWR) within the Environmental Influence on Early Aging (ENVIRONAGE) birth cohort framework.^28^ Furthermore, we investigate whether the identified cord blood proteins are associated with rapid growth over the first 12 months of life and with childhood weight, body mass index (BMI; calculated as weight in kilograms divided by height in meters squared) *z* score, waist circumference, and overweight (including obesity).

## Methods

This cohort study was approved by the ethical committees of East-Limburg Hospital and Hasselt University and was performed in accordance with the Helsinki Declaration. Mothers provided written informed consent at birth and follow-up. This study followed the Strengthening the Reporting of Observational Studies in Epidemiology (STROBE) reporting guideline.

### Study Population

The study was conducted within the ENVIRONAGE framework,^28^ an ongoing prospective birth cohort initiated in 2010, currently including more than 2200 mother-child pairs. In brief, mother-newborn pairs are recruited at arrival for delivery in the East-Limburg Hospital (Genk, Belgium) and followed-up longitudinally. Only singletons, mothers without cesarean delivery, and parents able to fill out a questionnaire in Dutch are eligible. At the first antenatal visit, maternal height and weight were measured, and maternal prepregnancy BMI was calculated. After delivery, the mother and child’s detailed lifestyle and sociodemographic information was gathered via questionnaires (eg, maternal age at delivery and education, smoking during pregnancy, parity, and race and ethnicity) and medical records (eg, newborn sex and date of delivery). Race and ethnicity were based on the native country of the newborn’s grandparents and described as European when 2 or more grandparents were European and non-European when at least 3 grandparents were not of European origin. Information on ethnicity was collected considering their potential role as a confounding factor stemming from genetic background.^29^ For this study, newborns were recruited between February 2010 and November 2017. Four to 6 years after the delivery, the mother-child pairs were contacted again to participate in the follow-up phase at Hasselt University (Diepenbeek, Belgium), where body anthropometric (ie, weight, height, and waist circumference) examinations, among others, were performed. A total of 1325 mother-child pairs were eligible for the follow-up phase before February 2022, of whom 588 participated (eFigure 1 in Supplement 1). Of these, a random subset with available cord blood was selected for the proteomics analysis (eTable 1 and eTable 2 in Supplement 1).

### Cord Blood Collection and Proteomics

Within 10 minutes after delivery, umbilical cord blood was gathered in BD Vacutainer plastic whole-blood tubes, spray-coated with K2EDTA (BD) and stored at −25 °C. A targeted proteomics analysis was performed on 300 EDTA plasma cord blood samples using Olink Explore (Olink Proteomics) (eMethods in Supplement 1). In brief, the levels of 370 proteins belonging to the Inflammation II Olink panel were examined using the proximity extension assay^30^ with next-generation sequencing readout (NovaSeq 6000; Illumina). Raw output data were quality controlled, normalized, and converted into normalized protein expressions (log_2_).^30^ Data normalization was performed using an internal extension control and an external plate control to adjust for intrarun and interrun variation. Observations more than 3 times the IQR below quartile 1 (Q1) or more than 3 time the IQR above Q3 were considered outliers. Two proteins (kininogen 1 and tumor necrosis factor superfamily member 9) and 12 cord blood samples did not pass the quality control criteria and were excluded from the analysis.

### Infant and Childhood Body Anthropometrics

Birth weight (grams) was measured with a digital scale (PF75 scale; CAE) equipped with a stadiometer to measure birth length (centimeters). The child’s BWR was calculated by dividing the birth weight by the birth weight reference, ie, the median birth weight of the population-specific reference growth curve considering parity, sex, and gestational age. This growth curve was calculated for Flanders by the Study Centre for Perinatal Epidemiology in 1997.^31^ Rapid postnatal growth was defined as the difference between the World Health Organization SD score of birth weight and estimated weight at 12 months greater than 0.67 SD, according to Ong et al.^32^ Sex- and age-specific estimated weight at 12 months was calculated via a 2-step estimation approach using fractional polynomials of age by sex.^33^ At the age of 4 to 6 years, children were followed up and weight (kilograms) and height (meters) were measured via an electronic scale (OMRON BF511) and upright stadiometer (Seca 213), respectively. BMI was calculated for children, and the BMI *z* score was formulated. Childhood overweight (including obesity) was defined based on the child BMI score greater than age- and sex-specific BMI cutoffs according to the International Obesity Task Force.^34^

### Statistical Analysis

All statistical analyses were performed using Rstudio version 4.2.3 (R Project for Statistical Computing). The study was performed in 3 steps. First, we executed a large cord blood proteomics analysis by fitting multiple linear regression models to assess the associations of 368 cord blood proteins with the infant’s birth weight and BWR, while accounting for a priori selected covariates: sex^3^ (male or female), gestational age^35^ (days), maternal prepregnancy BMI,^36^ maternal age at delivery^37^ (years), month and year of delivery,^38^ race and ethnicity^39^ (European or non-European), parity^40^ (first, second, third or more child), smoking during pregnancy^5^ (yes or no), and maternal education^9^ (low, middle, or high) (eMethods in Supplement 1). In a sensitivity analysis, we assessed whether diabetes gravidarum^6^ (yes or no), preeclampsia^7^ (yes or no), gestational hypertension^8^ (yes or no), paternal age at delivery,^41^ or paternal education^42^ (low, middle, or high) mediated the observed associations. We also ran the multiple linear regression models stratified by sex and linear regression models with an interaction term on a multiplicative scale between cord blood proteins and sex to examine whether the findings were sex-specific. Last, we performed a sensitivity analysis in which the linear regression models of BWR were not adjusted for sex, gestational age, or parity. Results are presented as estimates with 95% CIs for each doubling in proteins. A Bonferroni-adjusted 2-tailed *P* = .05 was used for statistical significance (eMethods in Supplement 1). Second, we assessed whether the birth weight–associated proteins were associated with rapid growth during the first 12 months of life using multiple logistic regression models, while accounting for the same covariates. Results are presented as odds ratios (ORs) with 95% CIs for each doubling in proteins. Third, we assessed whether the birth weight–associated proteins were associated with the child’s weight, BMI *z* score, waist circumference, or being overweight (including obesity) measured at ages 4 to 6 years, while accounting for the aforementioned covariates in addition to the age of the child (years) at the follow-up examination. Results are presented as estimates or ORs, with 95% CIs, for each doubling in proteins. Data were analyzed from February 2022 to September 2023.

## Results

### Population Characteristics

A total of 288 mother-infant pairs (125 [43.4%] male; mean [SD] gestation age, 277.2 [11.6] days) were included in analysis. Table 1 shows the body anthropometric and lifestyle characteristics of the participating mother-child pairs at birth and the follow-up examination. Mothers had a mean (SD) age of 30.4 (4.1) years at delivery, with mean (SD) prepregnancy BMI of 24.2 (4.3). For approximately half of the mothers (151 mothers [52.4%]), it was their first pregnancy. Most mothers did not smoke during pregnancy (265 mothers [92.0%]), did not have diabetes gravidarum (280 mothers [97.2%]), did not develop preeclampsia (284 mothers [98.6%]), and did not have gestational hypertension (278 mothers [99.7%]). Most mothers had attained a college or university degree (191 mothers [66.3%]). Among infants, mean (SD) birth weight was 3389.3 (492.9) grams and length was 50.2 (2.3) cm. The mean (SD) BWR was 1.0 (0.1), and 274 infants (95.1%) were of European descent. At age 12 months, 91 children (31.6%) had rapid growth. The mean (SD) age of the child at the follow-up examination was 4.6 (0.4) years. The mean (SD) weight at follow-up was 18.6 (2.6) kg, with BMI *z* score of 0.5 (0.8) and waist circumference of 53.0 (3.6) cm. At ages 4 to 6 years, 34 children (11.8%) were overweight or obese. Birth weight normalized for gestational age was positively associated with the BMI *z* score at age 4 to 6 years (*r* = 0.22; *P* = .002) (eFigure 2 in Supplement 1).

**Table 1.** Anthropometric and Lifestyle Characteristics of the Participating Mother-Child Pairs at Birth and the Follow-Up Examination

Characteristic	Participants, No. (%) (N = 288)
**Mother**	
Age at delivery, mean (SD), y	30.4 (4.1)
Prepregnancy BMI, mean (SD)	24.2 (4.3)
Parity	
First child	151 (52.4)
Second child	108 (37.5)
Third child	29 (10.1)
Smoking during pregnancy	23 (8.0)
Diabetes gravidarum	8 (2.8)
Preeclampsia	4 (1.4)
Gestational hypertension	10 (3.5)
Education^a^	
Low	18 (6.3)
Middle	79 (27.4)
High	191 (66.3)
**Child**	
Sex	
Boy	125 (43.4)
Girl	163 (56.6)
Gestational age, mean (SD), d	277.2 (11.6)
Birth weight, mean (SD), g	
Mean (SD)	3389.6 (492.9)
<2500	7 (2.4)
2500-4000	251 (87.2)
>4000	30 (10.4)
Birth length, mean (SD), cm	50.2 (2.3)
BWR, mean (SD)	1.0 (0.1)
Race and ethnicity^b^	
European	274 (95.1)
Not European	14 (4.9)
Rapid growth at age 12 mo	91 (31.6)
Age at follow-up, mean (SD), y	4.6 (0.4)
Weight at follow-up, mean (SD), kg	18.6 (2.6)
BMI *z* score at follow-up, mean (SD)	0.5 (0.8)
Waist circumference at follow-up, mean (SD), cm	53.0 (3.6)
Overweight (including obesity)	34 (11.8)

Abbreviations: BMI, body mass index (calculated as weight in kilograms divided by height in meters squared); BWR, birth weight ratio.

^a^ Maternal educational level was coded as low if the participant did not obtain a high school diploma, middle if the participant attained a high school diploma, and high if the participant attained a college or university degree.

^b^ Race and ethnicity were based on the native country of the newborn’s grandparents and described as European when 2 or more grandparents were European and non-European when at least 3 grandparents were not of European origin.

### Cord Blood Proteomics and Birth Outcomes

**Figure 1.**
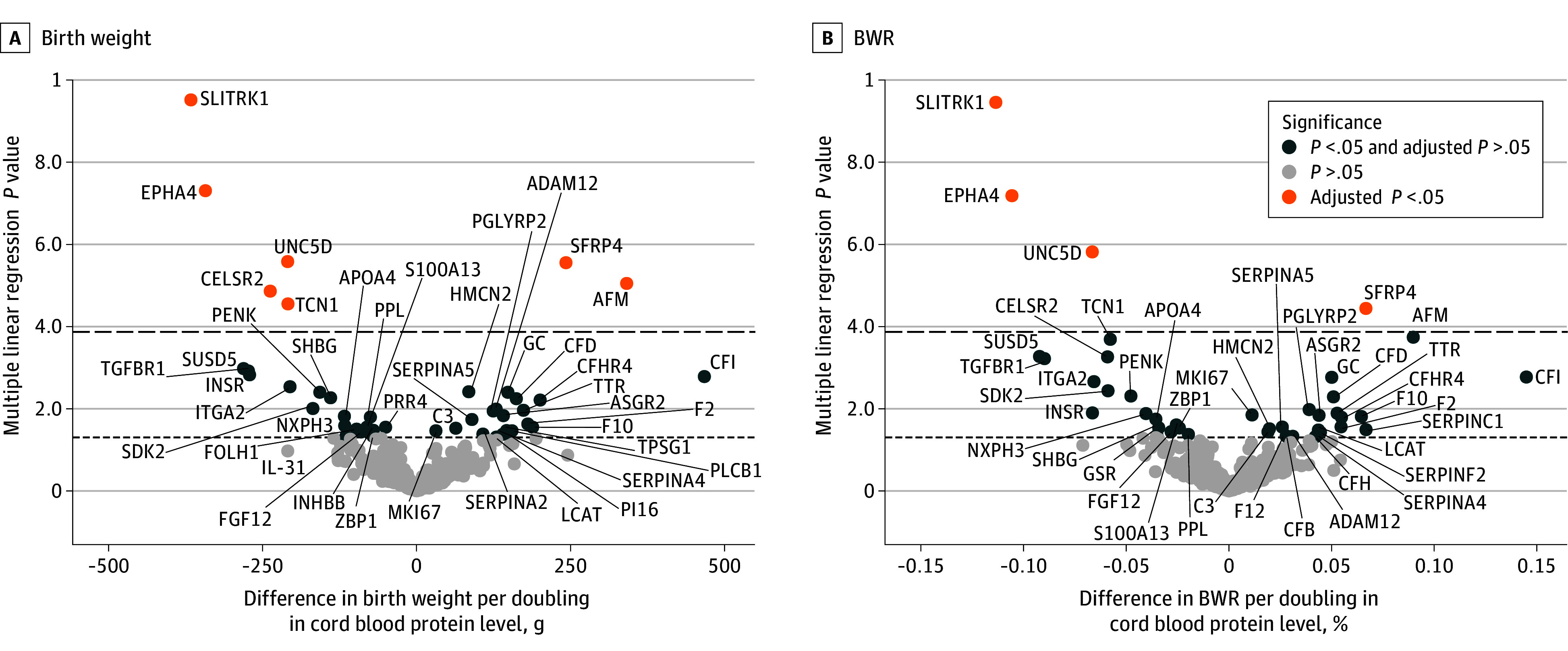
Volcano Plot of the Proteomics Analysis for Birth Weight and Birth Weight Ratio (BWR) Volcano plot of the results of the large proteomics analysis showing the difference in infant’s birth weight (grams) and BWR per doubling in cord blood protein levels (x-axis) vs multiple linear regression model *P* values (y-axis). Multiple linear regression models were adjusted for sex, gestational age, maternal prepregnancy body mass index, maternal age at delivery, month and year of delivery, ethnicity, parity, smoking during pregnancy, and maternal education. ADAM12 indicates ADAM metallopeptidase domain 12; AFM, afamin; APOA4, apolipoprotein A-IV; ASGR2, asialoglycoprotein receptor 2; C3, complement component 3; CELSR2, cadherin EGF LAG seven-pass G-type receptor 2; CFB, complement factor B; CFD, factor D; CFH, factor H; CFHR4, complement factor H-related protein 4; CFI, complement factor I; EPHA4, ephrin type-A receptor 4; F2, prothrombin; F10, factor X; F12, coagulation factor XII; FGF12, fibroblast growth factor 12; FOLH1, glutamate carboxypeptidase II; GC, human group-specific component; GSR, glutathione-disulfide reductase; HMCN2, hemicentin 2; IL-31, interleukin 31; INHBB, inhibin, beta B; INSR, insulin receptor; ITGA2, integrin alpha-2; LCAT, lecithin–cholesterol acyltransferase; MKI67, marker of proliferation Kiel 67; NXPH3, neurexophilin-3; PENK, proenkephalin; PGLYRP2, peptidoglycan recognition protein 2; PI16, peptidase inhibitor 16; PLCB1, phospholipase C beta 1; PPL, periplakin; PRR4, proline-rich protein 4; S100A13, S100 calcium-binding protein A13; SDK2, protein sidekick-2; SERPINA4, kallistatin; SERPINA5, serpina family A member 5; SERPINC1, serpin family C member 1; SERPINF2, serpin family F member 2; SFRP4, secreted frizzled-related protein 4; SHBG, sex hormone–binding globulin; SLITRK1, SLIT and NTRK-like protein 1; SUSD5, sushi domain-containing protein 5; TCN1, transcobalamin; TGFBR1, transforming growth factor-beta receptor 1; TPSG1, tryptase gamma 1; TTR, transthyretin; UNC5D, unc-5 netrin receptor D; and ZBP1, Z-DNA binding protein 1.

In our large cord blood proteomics analysis, 368 cord blood proteins were regressed on birth weight and BWR, resulting in 44 birth weight–associated and 42 BWR-associated proteins (eTable 3 in Supplement 1). After multiple testing correction with Bonferroni, 7 proteins remained significantly associated with birth weight and 4 proteins remained significantly associated with BWR (Figure 1). The interprotein correlations among the 7 statistically significant proteins (absolute *r* range, 0.06 to 0.78) are shown in eFigure 3 in Supplement 1. Afamin was positively associated with birth weight (coefficient, 341.16 [95% CI, 192.76 to 489.50] g), while secreted frizzled-related protein 4 (SFRP4) was positively associated with both birth weight (coefficient, 242.60 [95% CI, 142.77 to 342.43] g) and BWR (coefficient, 0.07 [95% CI, 0.04 to 0.10]). Negatively associated proteins were cadherin EGF LAG 7-pass G-type receptor 2 (CELSR2; birth weight: coefficient, −237.52 [95% CI, −343.15 to −131.89] g), ephrin type-A receptor 4 (EPHA4; birth weight: coefficient, −342.78 [95% CI, −463.10 to −222.47] g; BWR: coefficient, −0.11 [95% CI, −0.14 to −0.07]), SLIT and NTRK-like protein 1 (SLITRK1; birth weight: coefficient, −366.32 [95% CI, −476.66 to −255.97] g; BWR: coefficient, −0.11 [95% CI, −0.15 to −0.08]), transcobalamin-1 (TCN1; birth weight: coefficient, −208.75 [95% CI, −305.23 to −112.26] g), and netrin receptor UNC5D (UNC5D; birth weight: coefficient, −209.27 [95% CI, −295.14 to −123.40] g; BWR: coefficient, −0.07 [95% CI, −0.09 to −0.04]). eTable 4 in Supplement 1 shows the numeric data for the associations between cord blood protein levels and birth weight and the BWR. In sensitivity analyses, we found that excluding mothers with diabetes gravidarum, preeclampsia, or gestational hypertension or additionally adjusting for paternal age at delivery, paternal education, or all other proteins that were statistically significantly associated with birth weight or BWR did not significantly change the observed associations. Furthermore, the results for BWR remained the same when the multiple linear regression models were not adjusted for sex, gestational age, and parity (eTable 5 in Supplement 1). In addition, no associations were sex-specific (eTable 5 and eTable 6 in Supplement 1). The associations between all the measured proteins and the infant’s birth weight and BWR are depicted in eTable 3 in Supplement 1.

### Cord Blood Proteins and Rapid Growth at Age 12 Months

We assessed whether the birth weight–associated cord blood proteins were associated with rapid growth during the first 12 months (Table 2). Two of 7 proteins were associated with rapid growth: a doubling in cord blood afamin was associated with lower odds of rapid growth (OR, 0.32 [95% CI, 1.11 to 0.88]), while a doubling in TCN1 was associated with higher odds (OR, 2.44 [95% CI, 1.26 to 4.80]).

**Table 2.** Cord Blood Proteins and the Odds for Rapid Growth at 12 Months and Overweight at Ages 4 to 6 Years

Cord blood protein	Odds ratio per doubling in cord blood protein level (95% CI)^a^	
	Rapid growth at age 12 mo^b^	Overweight at age 4-6 y
AFM	0.32 (0.11-0.88)	0.19 (0.05-0.70)
CELSR2	1.39 (0.67-2.89)	0.51 (0.19-1.33)
EPHA4	1.12 (0.49-2.60)	0.49 (0.15-1.62)
SFRP4	0.49 (0.29-1.01)	2.24 (0.98-3.27)
SLITRK1	1.30 (0.60-2.84)	0.32 (0.10-0.99)
TCN1	2.44 (1.26-4.80)	0.53 (0.20-1.37)
UNC5D	0.92 (0.51-1.65)	0.59 (0.25-1.39)

Abbreviations: AFM, afamin; CELSR2, cadherin EGF LAG seven-pass G-type receptor 2; EPHA4, ephrin type-A receptor 4; SFRP4, secreted frizzled-related protein 4; SLITRK1, SLIT and NTRK-like protein 1; TCN1, transcobalamin-1; UNC5D, unc-5 netrin receptor D.

^a^ Multiple logistic regression models were adjusted for sex, gestational age, maternal prepregnancy body mass index, maternal age at delivery, month and year of delivery, race and ethnicity, parity, smoking during pregnancy, maternal education, birth weight (only for overweight), and age of the child at the follow-up examination (only for overweight).

^b^ Rapid growth is defined by the World Health Organization and overweight by the International Obesity Taskforce.

### Cord Blood Proteins and Weight, BMI *z* Score, Waist Circumference, and Overweight at Ages 4 to 6 Years

**Figure 2.**
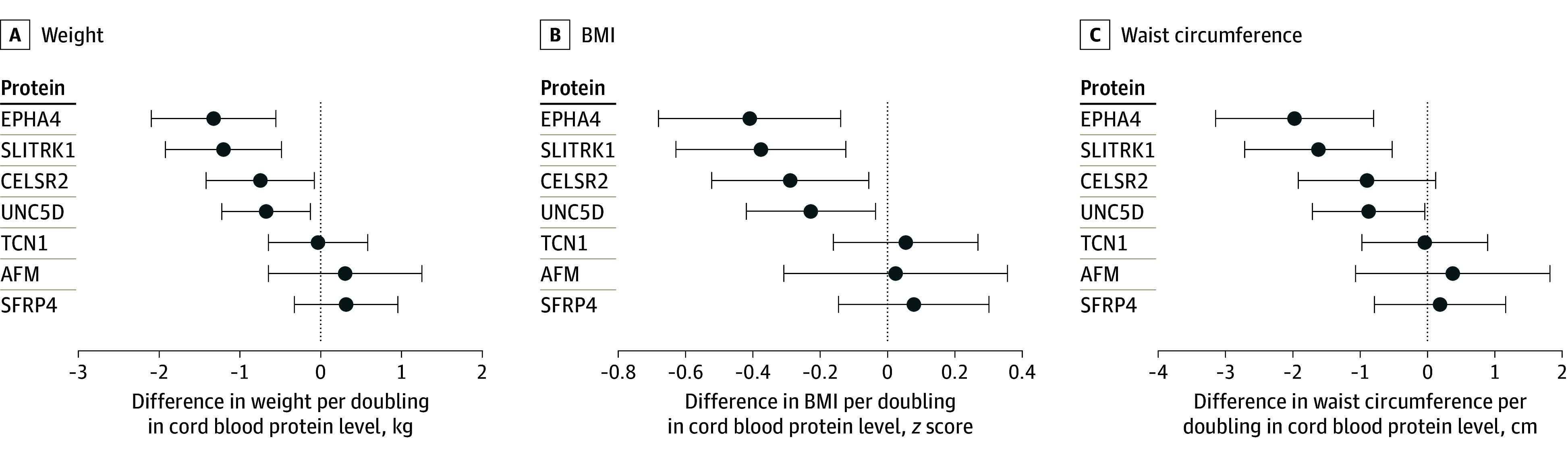
Forest Plot of the Child’s Weight, Body Mass Index (BMI) *z* Score, and Waist Circumference With Cord Blood Proteins Differences (with 95% CIs) were calculated at age 4 to 6 years per doubling in cord blood protein levels. Multiple linear regression models were adjusted for sex, gestational age, maternal prepregnancy BMI (calculated as weight in kilograms divided by height in meters squared), maternal age at delivery, month and year of delivery, race and ethnicity, parity, smoking during pregnancy, maternal education, and age of the child at the follow-up examination. Corresponding numeric data are provided in eTable 7 in Supplement 1. AFM indicates afamin; CELSR2, cadherin EGF LAG seven-pass G-type receptor 2; EPHA4, ephrin type-A receptor 4; SFRP4, secreted frizzled-related protein 4; SLITRK1, SLIT and NTRK-like protein 1; TCN1, transcobalamin-1; UNC5D, unc-5 netrin receptor D.

In a final analysis, we evaluated whether the identified cord blood proteomic markers of birth weight were associated with child body anthropometrics (weight, BMI *z* score, waist circumference, and overweight). Of 7 cord blood proteins, 4 were negatively associated with weight, 4 with BMI *z* score, 3 with waist circumference, and 2 with overweight: a doubling in cord blood afamin was associated with lower odds of overweight (OR, 0.19 [95% CI, 0.05 to 0.70]); a doubling in CELSR2 was associated with lower weight (coefficient, −0.75 [95% CI, −1.42 to −0.08] g) and BMI *z* score (coefficient, −0.29 [95% CI, −0.52 to −0.06]); a doubling in EPHA4 was associated with lower weight (coefficient, −1.33 [95% CI, −2.10 to −0.55] g), BMI *z* score (coefficient, −0.41 [95% CI, −0.52 to −0.06]), and waist circumference (coefficient, −1.98 [95% CI, −2.10 to −0.55] cm); a doubling in SLITRK1 was associated with lower weight (coefficient, −1.20 [95% CI, −1.92 to −0.48] g), BMI *z* score (coefficient, −0.38 [95% CI, −0.63 to −0.12]), and waist circumference (coefficient, −1.62 [95% CI, −2.72 to −0.52] cm) and lower odds of overweight (OR, 0.32 [95% CI, 0.10 to 0.99]); and a doubling in UNC5D was associated with lower weight (coefficient, −0.68 [95% CI, −1.22 to −0.13] g), BMI *z* score (coefficient, −0.23 [95% CI, −0.42 to −0.04]), and waist circumference (coefficient, −0.87 [95% CI, −1.71 to −0.04] cm) (Table 2 and Figure 2).

## Discussion

In this cohort study, we present the first extensive analysis of cord blood proteomics, to our knowledge, encompassing 368 cord blood proteins and birth weight. Furthermore, we studied cord blood proteomic signatures associated with birth weight and their implications for growth trajectories in infancy and early childhood. We found that higher cord blood protein levels of afamin and SFRP4 and lower cord blood levels of CELSR2, EPHA4, SLITRK1, TCN1, and UNC5D were associated with birth weight and/or BWR. Of the 7 birth weight–associated cord blood proteins, we observed that afamin and TCN1 levels were associated with lower and higher odds for rapid growth at 12 months old, respectively. Furthermore, the cord blood levels of 5 proteins (afamin, CELSR2, EPHA4, SLITRK1, and UNC5D) were negatively associated with at least 1 of the growth-related parameters measured at ages 4 to 6 years: weight, BMI *z* score, waist circumference, and overweight. The 7 aforementioned proteins might be associated with birth weight and early life growth trajectories via different mechanisms (eg, regulating growth hormone synthesis, metabolism and metabolic disorders, embryonic development, and neurological pathways), although their biological function has rarely been studied in cord blood.

### Growth Hormones

Previous research showed that cell surface EPHA4 can increase insulin-like growth factor 1 (IGF1) production via receptor signaling.^43,44^ IGF1 is synthesized by the mother, placenta, and fetus during pregnancy and is essential for the growth and development of the placenta.^45^ Positive correlations have been observed between cord blood serum IGF1 levels and birth weight (*r* = 0.67).^46^ Furthermore, *Epha4* knockout mice had a shorter stature and lower weight,^43^ indicating that EPHA4 levels might influence postnatal body growth. Nevertheless, the biological function of human plasma EPHA4 levels has not been examined comprehensively and hence, we are the first, to our knowledge, to report negative associations with (birth) weight and body anthropometrics.

### Metabolism and Metabolic Disorders

Of the 9 cord blood proteins associated with birth weight and body anthropometrics in this study, 3 are associated with metabolism or metabolic disorders. High serum whole blood afamin and SFRP4 concentrations in the first trimester have been associated with the onset of gestational diabetes.^47,48^ Both afamin overexpression in transgenic mice^49^ and gestational diabetes in humans^49,50,51^ have been associated with a higher birth weight and/or increased risk of large for gestational age, which is in line with the findings of this study. Nevertheless, since no other study that we know of considered afamin and SFRP4 levels in cord blood, our findings are novel and require further validation. In addition, it should be noted that excluding mothers with diabetes gravidarum from the analyses did not significantly change the observed associations.

Vitamin B_12_ is involved in the production of fatty acids and amino acids^52^ and required for a healthy fetal growth, as maternal vitamin B_12_ deficiency has been associated with low birth weight and intrauterine growth retardation.^53,54,55^ TCN1 binds up to 70% of the vitamin B_12_ transported across the placenta, and positive associations have been observed between cord blood vitamin B_12_ levels and placental TCN1 protein abundance.^53^ To our knowledge, no studies have examined the direct association between cord blood TCN1 levels and birth weight or early life growth trajectories, and we are the first study, to our knowledge, to report a negative association between both.

### Wnt Signaling Pathway

Afamin and SFRP4, 2 proteins positively associated with birth weight, have been linked to the Wnt signaling pathway.^56,57,58^ This signal transduction pathway is involved in cell proliferation, differentiation, survival, migration, and polarity^56^ during embryonic and postnatal development. Its main functions are body-axis formation and organ development.^59^ While afamin functions as an extracellular chaperone for poorly soluble, acylated Wnt-proteins, such as Wnt family member 3A, to help them bind to their receptor,^58^ SFRP4 operates as an extracellular antagonist to balance Wnt function in bone development, for example.^57^ Literature studying the association between the Wnt signaling pathway and birth weight is scarce; 1 study reported high Wnt2 gene methylation in placental tissue in association with low birth weight percentile in 16 neonates.^60^

### Neurological Pathways

Our findings on afamin, CELSR2, SLITRK1, and UNC5D need further evaluation, but during fetal development and postnatally, they are involved in neurological pathways. Specifically, afamin has a neuroprotective association, as it transports vitamin E across the blood-brain barrier.^61^ In in vitro settings, both afamin and vitamin E enhance cortical neuronal survival.^62^ Second, CELSR2 has roles in epithelial planar cell polarity and ciliogenesis.^63^ CELSR2-deficient mice had a poor cerebrospinal fluid circulation and hydrocephalus.^64^ To our knowledge, no literature is available on the association of cord blood CELSR2 levels with birth weight and childhood anthropometrics, making our findings novel. Third, SLITRK1 regulates the synapse formation between hippocampal neurons and neuronal survival.^65,66^ To date, only 1 study found that *Slitrk1* knockout mice had a lower body weight during postnatal development.^67^ Last, UNC5D is a netrin receptor that promotes the outgrowth and guidance of spinal axons toward the floor plate during embryogenesis in vertebrates.^68^ An epidemiological study in 80 mother-newborn pairs^69^ showed that maternal netrin-4 blood levels decreased as birth weight centiles increased for fetuses with an intrauterine growth restriction. These results might be in line with our findings. Nevertheless, the exact role of UNC5D in weight and growth needs to be examined.

### Strengths and Limitations

Our study has several strengths. First, the study is situated in a prospective birth cohort where weight and body anthropometrics at birth, infancy, and early childhood could be evaluated in associated with cord blood proteins. Second, we were able to correct for a large number of potential confounders and performed sensitivity analyses that showed the robustness of our observed associations. Third, our study population was representative for the gestational segment of Flanders population.^28^ Fourth, we measured a large number of proteins with a high reliability using the proximity extension assay technique.

We acknowledge some study limitations. First, our study was not a proteome-wide association study, as a targeted protein panel was used. Second, to reduce the chance of false-positive findings, the reported *P* values were Bonferroni adjusted. Nevertheless, since 368 cord blood proteins were regressed on birth weight and BWR, there is a small opportunity to detect features unique to this dataset. Third, the study consisted of a relatively small number of mother-child pairs, which prevents detailed subgroup analyses.

## Conclusions

This cohort study investigated the associations among 368 inflammatory-related cord blood proteins and birth weight and early life growth trajectories, as the cord blood proteome is associated with the physiological and pathological status of the fetus. We found robust cord blood proteomic signatures associated with birth weight, and most of these proteins were still associated with weight and growth early in life. Cord blood proteins associated with birth weight and growth in early life may be due to a variety of proposed mechanisms, including growth hormone synthesis, metabolism and metabolic disorders, neurological pathways, and placental vascularization. Overall, our findings suggest that stressors that could affect the cord blood proteome during pregnancy might have long-lasting associations with weight and body anthropometrics and possibly even be associated with disease development later in life.

## Notes

Supplement 1. **eMethods.** Covariables, Olink Proteomics, and Bonferroni Correction **eTable 1**. Main Anthropometric and Lifestyle Characteristics of the Participating Mother-Child Pairs Compared With Those Who Participated in the Follow-Up Examination Before February 2022 **eTable 2.** Main Anthropometric and Lifestyle Characteristics of the Participating Mother-Child Pairs Compared With Those of all Mother-Child Pairs Eligible for Follow-Up Between October 2014 and February 2022 **eTable 3.** Multiple Linear Regression Models Examining the Association of Cord Blood Proteins With Birth Weight or BWR in the Large Proteomics Analysis **eTable 4.** Numeric Data of the Associations Between Cord Blood Proteins and the Infant’s Birth Weight and BWR **eTable 5.** Sensitivity Analyses of Associations of Cord Blood Proteins With Infant Body Weight or BWR After Exclusion of Diabetes Gravidarum, Preeclampsia, or Gestational Hypertension, or After Additional Adjustment for Paternal Age at Delivery, Paternal Education, or All Other Proteins Statistically Significantly Associated With Birth Weight or BWR, or Stratified by Sex **eTable 6.** Sensitivity Analyses of Associations of Cord Blood Proteins With Infant Body Weight or BWR, With an Interaction Term Between Cord Blood Protein Levels and Sex **eTable 7.** Numeric Data of the Associations of Child Weight, BMI *z* Score, or Waist Circumference With Cord Blood Proteins **eFigure 1.** Participant Flowchart Depicting the Selection of Participants Enrolled in the ENVIRONAGE Birth Cohort for Arriving at the Final Study Sample Size **eFigure 2.** Scatterplot Showing the Correlation Between the BMI *z* Score at 4 to 6 Years Old and Birth Weight Normalized for Gestational Age **eFigure 3.** Pearson Correlation Matrices Between the Proteins Statistically Significant Associated With Birth Weight and/or BWR in the Large Proteomics Analysis
eReferences.

Supplement 2.
Data Sharing Statement

## References

[1] Zhang Y, Li H, Liu SJ, . The associations of high birth weight with blood pressure and hypertension in later life: a systematic review and meta-analysis. Hypertens Res. 2013;36(8):725-735. doi:10.1038/hr.2013.33 23595042

[2] Vilanova CS, Hirakata VN, de Souza Buriol VC, Nunes M, Goldani MZ, da Silva CH. The relationship between the different low birth weight strata of newborns with infant mortality and the influence of the main health determinants in the extreme south of Brazil. Popul Health Metr. 2019;17(1):15. doi:10.1186/s12963-019-0195-7 31775758 PMC6882357

[3] Calais-Ferreira L, Barreto ME, Mendonça E, . Birthweight, gestational age and familial confounding in sex differences in infant mortality: a matched co-twin control study of Brazilian male-female twin pairs identified by population data linkage. Int J Epidemiol. 2022;51(5):1502-1510. doi:10.1093/ije/dyab242 34849953 PMC9557851

[4] Kjøllesdal MKR, Holmboe-Ottesen G. Dietary patterns and birth weight—a review. AIMS Public Health. 2014;1(4):211-225. doi:10.3934/Publichealth.2014.4.211 29546087 PMC5690254

[5] Kataoka MC, Carvalheira APP, Ferrari AP, Malta MB, de Barros Leite Carvalhaes MA, de Lima Parada CMG. Smoking during pregnancy and harm reduction in birth weight: a cross-sectional study. BMC Pregnancy Childbirth. 2018;18(1):67. doi:10.1186/s12884-018-1694-4 29530015 PMC5848535

[6] Persson M, Shah PS, Rusconi F, ; International Network for Evaluating Outcomes of Neonates. Association of maternal diabetes with neonatal outcomes of very preterm and very low-birth-weight infants: an international cohort study. JAMA Pediatr. 2018;172(9):867-875. doi:10.1001/jamapediatrics.2018.1811 29971428 PMC6143059

[7] Gunnarsdottir J, Cnattingius S, Lundgren M, Selling K, Högberg U, Wikström AK. Prenatal exposure to preeclampsia is associated with accelerated height gain in early childhood. PLoS One. 2018;13(2):e0192514. doi:10.1371/journal.pone.0192514 29438394 PMC5811001

[8] Lau TK, Pang MW, Sahota DS, Leung TN. Impact of hypertensive disorders of pregnancy at term on infant birth weight. Acta Obstet Gynecol Scand. 2005;84(9):875-877. doi:10.1111/j.0001-6349.2005.00740.x 16097979

[9] Parker JD, Schoendorf KC, Kiely JL. Associations between measures of socioeconomic status and low birth weight, small for gestational age, and premature delivery in the United States. Ann Epidemiol. 1994;4(4):271-278. doi:10.1016/1047-2797(94)90082-5 7921316

[10] Cogswell ME, Yip R. The influence of fetal and maternal factors on the distribution of birthweight. Semin Perinatol. 1995;19(3):222-240. doi:10.1016/S0146-0005(05)80028-X 7570074

[11] Chandrasekaran N. Induction of labor for a suspected large-for-gestational-age/macrosomic fetus. Best Pract Res Clin Obstet Gynaecol. 2021;77:110-118. doi:10.1016/j.bpobgyn.2021.09.005 34602354

[12] Cutland CL, Lackritz EM, Mallett-Moore T, ; Brighton Collaboration Low Birth Weight Working Group. Low birth weight: case definition & guidelines for data collection, analysis, and presentation of maternal immunization safety data. Vaccine. 2017;35(48 Pt A):6492-6500. doi:10.1016/j.vaccine.2017.01.049 29150054 PMC5710991

[13] Jana A, Saha UR, Reshmi RS, Muhammad T. Relationship between low birth weight and infant mortality: evidence from National Family Health Survey 2019-21, India. Arch Public Health. 2023;81(1):28. doi:10.1186/s13690-023-01037-y 36803539 PMC9942291

[14] Magnusson Å, Laivuori H, Loft A, . The association between high birth weight and long-term outcomes-implications for assisted reproductive technologies: a systematic review and meta-analysis. Front Pediatr. 2021;9:675775. doi:10.3389/fped.2021.675775 34249812 PMC8260985

[15] Schellong K, Schulz S, Harder T, Plagemann A. Birth weight and long-term overweight risk: systematic review and a meta-analysis including 643,902 persons from 66 studies and 26 countries globally. PLoS One. 2012;7(10):e47776. doi:10.1371/journal.pone.0047776 23082214 PMC3474767

[16] Baran J, Weres A, Czenczek-Lewandowska E, Leszczak J, Kalandyk-Osinko K, Mazur A. Relationship between children’s birth weight and birth length and a risk of overweight and obesity in 4-15-year-old children. Medicina (Kaunas). 2019;55(8):487. doi:10.3390/medicina55080487 31443282 PMC6722569

[17] Must A, Spadano J, Coakley EH, Field AE, Colditz G, Dietz WH. The disease burden associated with overweight and obesity. JAMA. 1999;282(16):1523-1529. doi:10.1001/jama.282.16.1523 10546691

[18] Mandy M, Nyirenda M. Developmental origins of health and disease: the relevance to developing nations. Int Health. 2018;10(2):66-70. doi:10.1093/inthealth/ihy006 29528398 PMC5856182

[19] Suski M, Bokiniec R, Szwarc-Duma M, . Prospective plasma proteome changes in preterm infants with different gestational ages. Pediatr Res. 2018;84(1):104-111. doi:10.1038/s41390-018-0003-2 29795197

[20] Hedman AM, Lundholm C, Andolf E, Pershagen G, Fall T, Almqvist C. Longitudinal plasma inflammatory proteome profiling during pregnancy in the Born Into Life study. Sci Rep. 2020;10(1):17819. doi:10.1038/s41598-020-74722-5 33082373 PMC7575597

[21] Zaghlool SB, Sharma S, Molnar M, . Revealing the role of the human blood plasma proteome in obesity using genetic drivers. Nat Commun. 2021;12(1):1279. doi:10.1038/s41467-021-21542-4 33627659 PMC7904950

[22] Assi E, D’Addio F, Mandò C, . Placental proteome abnormalities in women with gestational diabetes and large-for-gestational-age newborns. BMJ Open Diabetes Res Care. 2020;8(2):e001586. doi:10.1136/bmjdrc-2020-001586 33188009 PMC7668299

[23] Liu CW, Bramer L, Webb-Robertson BJ, Waugh K, Rewers MJ, Zhang Q. Temporal profiles of plasma proteome during childhood development. J Proteomics. 2017;152:321-328. doi:10.1016/j.jprot.2016.11.016 27890796 PMC5219852

[24] Siino V, Ali A, Accardi G, . Plasma proteome profiling of healthy individuals across the life span in a Sicilian cohort with long-lived individuals. Aging Cell. 2022;21(9):e13684. doi:10.1111/acel.13684 35932462 PMC9470904

[25] Papathanasiou AE, Briana DD, Gavrili S, . Cord blood fatty acid-binding protein-4 levels are upregulated at both ends of the birthweight spectrum. Acta Paediatr. 2019;108(11):2083-2088. doi:10.1111/apa.14826 31025416

[26] Senses DA, Coskun A, Kiseli M, . Is there a relationship between cord blood pregnancy-associated plasma protein-A and birth weight and length? Early Hum Dev. 2007;83(7):479-482. doi:10.1016/j.earlhumdev.2006.10.005 17161560

[27] Song HJ, Zhang P, Guo XJ, . The proteomic analysis of human neonatal umbilical cord serum by mass spectrometry. Acta Pharmacol Sin. 2009;30(11):1550-1558. doi:10.1038/aps.2009.140 19890362 PMC4003003

[28] Janssen BG, Madhloum N, Gyselaers W, . Cohort profile: The Environmental Influence on Early Ageing (ENVIRONAGE): a birth cohort study. Int J Epidemiol. 2017;46(5):1386-1387m. doi:10.1093/ije/dyx033 28089960

[29] Sleurs H, Silva AI, Bijnens EM, . Exposure to Residential Green Space and Bone Mineral Density in Young Children. JAMA Netw Open. 2024;7(1):e2350214. doi:10.1001/jamanetworkopen.2023.50214 38175647 PMC10767584

[30] Assarsson E, Lundberg M, Holmquist G, . Homogenous 96-plex PEA immunoassay exhibiting high sensitivity, specificity, and excellent scalability. PLoS One. 2014;9(4):e95192. doi:10.1371/journal.pone.0095192 24755770 PMC3995906

[31] Vleugels A, Bekaert A. The Flemish Centre for the Study of Perinatal Epidemiology and its registry. Qual Assur Health Care. 1992;4(2):115-124. 1511145

[32] Ong KKL, Ahmed ML, Emmett PM, Preece MA, Dunger DB. Association between postnatal catch-up growth and obesity in childhood: prospective cohort study. BMJ. 2000;320(7240):967-971. doi:10.1136/bmj.320.7240.967 10753147 PMC27335

[33] Handakas E, Keski-Rahkonen P, Chatzi L, . Cord blood metabolic signatures predictive of childhood overweight and rapid growth. Int J Obes (Lond). 2021;45(10):2252-2260. doi:10.1038/s41366-021-00888-1 34253844 PMC8455328

[34] Cole TJ, Bellizzi MC, Flegal KM, Dietz WH. Establishing a standard definition for child overweight and obesity worldwide: international survey. BMJ. 2000;320(7244):1240-1243. doi:10.1136/bmj.320.7244.1240 10797032 PMC27365

[35] Gutbrod T, Wolke D, Soehne B, Ohrt B, Riegel K. Effects of gestation and birth weight on the growth and development of very low birthweight small for gestational age infants: a matched group comparison. Arch Dis Child Fetal Neonatal Ed. 2000;82(3):F208-F214. doi:10.1136/fn.82.3.F208 10794788 PMC1721075

[36] Liu L, Ma Y, Wang N, Lin W, Liu Y, Wen D. Maternal body mass index and risk of neonatal adverse outcomes in China: a systematic review and meta-analysis. BMC Pregnancy Childbirth. 2019;19(1):105. doi:10.1186/s12884-019-2249-z 30922244 PMC6440121

[37] Wang S, Yang L, Shang L, . Changing trends of birth weight with maternal age: a cross-sectional study in Xi’an city of Northwestern China. BMC Pregnancy Childbirth. 2020;20(1):744. doi:10.1186/s12884-020-03445-2 33256654 PMC7708914

[38] Currie J, Schwandt H. Within-mother analysis of seasonal patterns in health at birth. Proc Natl Acad Sci U S A. 2013;110(30):12265-12270. doi:10.1073/pnas.1307582110 23836632 PMC3725083

[39] Morisaki N, Kawachi I, Oken E, Fujiwara T. Social and anthropometric factors explaining racial/ethnical differences in birth weight in the United States. Sci Rep. 2017;7:46657. doi:10.1038/srep46657 28429791 PMC5399358

[40] Shah PS; Knowledge Synthesis Group on Determinants of LBW/PT births. Parity and low birth weight and preterm birth: a systematic review and meta-analyses. Acta Obstet Gynecol Scand. 2010;89(7):862-875. doi:10.3109/00016349.2010.486827 20583931

[41] Reichman NE, Teitler JO. Paternal age as a risk factor for low birthweight. Am J Public Health. 2006;96(5):862-866. doi:10.2105/AJPH.2005.066324 16571696 PMC1470584

[42] Kehinde OA, Njokanma OF, Olanrewaju DM. Parental socioeconomic status and birth weight distribution of Nigerian term newborn babies. Niger J Paediatr. 2013;40:299-302.

[43] Jing X, Miyajima M, Sawada T, . Crosstalk of humoral and cell-cell contact-mediated signals in postnatal body growth. Cell Rep. 2012;2(3):652-665. doi:10.1016/j.celrep.2012.08.021 22999939

[44] Sawada T, Arai D, Jing X, Miyajima M, Frank SJ, Sakaguchi K. Molecular interactions of EphA4, growth hormone receptor, Janus kinase 2, and signal transducer and activator of transcription 5B. PLoS One. 2017;12(7):e0180785. doi:10.1371/journal.pone.0180785 28686668 PMC5501605

[45] Hiden U, Glitzner E, Hartmann M, Desoye G. Insulin and the IGF system in the human placenta of normal and diabetic pregnancies. J Anat. 2009;215(1):60-68. doi:10.1111/j.1469-7580.2008.01035.x 19467150 PMC2714639

[46] Osorio M, Torres J, Moya F, . Insulin-like growth factors (IGFs) and IGF binding proteins-1, -2, and -3 in newborn serum: relationships to fetoplacental growth at term. Early Hum Dev. 1996;46(1-2):15-26. doi:10.1016/0378-3782(96)01737-9 8899351

[47] Köninger A, Mathan A, Mach P, . Is afamin a novel biomarker for gestational diabetes mellitus: a pilot study. Reprod Biol Endocrinol. 2018;16(1):30. doi:10.1186/s12958-018-0338-x 29587878 PMC5870691

[48] Schuitemaker JHN, Beernink RHJ, Franx A, Cremers TIFH, Koster MPH. First trimester secreted frizzled-related protein 4 and other adipokine serum concentrations in women developing gestational diabetes mellitus. PLoS One. 2020;15(11):e0242423. doi:10.1371/journal.pone.0242423 33206702 PMC7673552

[49] Kronenberg F, Kollerits B, Kiechl S, . Plasma concentrations of afamin are associated with the prevalence and development of metabolic syndrome. Circ Cardiovasc Genet. 2014;7(6):822-829. doi:10.1161/CIRCGENETICS.113.000654 25176938

[50] Yang Y, Wang Z, Mo M, . The association of gestational diabetes mellitus with fetal birth weight. J Diabetes Complications. 2018;32(7):635-642. doi:10.1016/j.jdiacomp.2018.04.008 29907325

[51] He LR, Yu L, Guo Y. Birth weight and large for gestational age trends in offspring of pregnant women with gestational diabetes mellitus in southern China, 2012-2021. Front Endocrinol (Lausanne). 2023;14:1166533. doi:10.3389/fendo.2023.1166533 37214242 PMC10194652

[52] Allen LH. Vitamin B-12. Adv Nutr. 2012;3(1):54-55. doi:10.3945/an.111.001370 22332101 PMC3262614

[53] Layden AJ, O’Brien KO, Pressman EK, Cooper EM, Kent TR, Finkelstein JL. Vitamin B_12_ and placental expression of transcobalamin in pregnant adolescents. Placenta. 2016;45:1-7. doi:10.1016/j.placenta.2016.06.011 27577703

[54] Dwarkanath P, Barzilay JR, Thomas T, Thomas A, Bhat S, Kurpad AV. High folate and low vitamin B-12 intakes during pregnancy are associated with small-for-gestational age infants in South Indian women: a prospective observational cohort study. Am J Clin Nutr. 2013;98(6):1450-1458. doi:10.3945/ajcn.112.056382 24108785

[55] Muthayya S, Kurpad AV, Duggan CP, . Low maternal vitamin B_12_ status is associated with intrauterine growth retardation in urban South Indians. Eur J Clin Nutr. 2006;60(6):791-801. doi:10.1038/sj.ejcn.1602383 16404414

[56] Guo R, Xing QS. Roles of Wnt signaling pathway and ROR2 receptor in embryonic development: an update review article. Epigenet Insights. Published online January 31, 2022. doi:10.1177/25168657211064232 35128307 PMC8808015

[57] Haraguchi R, Kitazawa R, Mori K, . sFRP4-dependent Wnt signal modulation is critical for bone remodeling during postnatal development and age-related bone loss. Sci Rep. 2016;6:25198. doi:10.1038/srep25198 27117872 PMC4846872

[58] Naschberger A, Orry A, Lechner S, . Structural evidence for a role of the multi-functional human glycoprotein afamin in Wnt Transport. Structure. 2017;25(12):1907-1915.e5. doi:10.1016/j.str.2017.10.006 29153507

[59] Hikasa H, Sokol SY. Wnt signaling in vertebrate axis specification. Cold Spring Harb Perspect Biol. 2013;5(1):a007955. doi:10.1101/cshperspect.a007955 22914799 PMC3579404

[60] Ferreira JC, Choufani S, Grafodatskaya D, . WNT2 promoter methylation in human placenta is associated with low birthweight percentile in the neonate. Epigenetics. 2011;6(4):440-449. doi:10.4161/epi.6.4.14554 21474991

[61] Jerkovic L, Voegele AF, Chwatal S, . Afamin is a novel human vitamin E-binding glycoprotein characterization and in vitro expression. J Proteome Res. 2005;4(3):889-899. doi:10.1021/pr0500105 15952736

[62] Heiser M, Hutter-Paier B, Jerkovic L, . Vitamin E binding protein afamin protects neuronal cells in vitro. J Neural Transm Suppl. 2002;62(Suppl):337-345. doi:10.1007/978-3-7091-6139-5_32 12456077

[63] Basta LP, Sil P, Jones RA, Little KA, Hayward-Lara G, Devenport D. Celsr1 and Celsr2 exhibit distinct adhesive interactions and contributions to planar cell polarity. Front Cell Dev Biol. 2023;10:1064907. doi:10.3389/fcell.2022.1064907 36712970 PMC9878842

[64] Tissir F, Qu Y, Montcouquiol M, . Lack of cadherins Celsr2 and Celsr3 impairs ependymal ciliogenesis, leading to fatal hydrocephalus. Nat Neurosci. 2010;13(6):700-707. doi:10.1038/nn.2555 20473291

[65] Beaubien F, Raja R, Kennedy TE, Fournier AE, Cloutier JF. Slitrk1 is localized to excitatory synapses and promotes their development. Sci Rep. 2016;6:27343. doi:10.1038/srep27343 27273464 PMC4895136

[66] Proenca CC, Gao KP, Shmelkov SV, Rafii S, Lee FS. Slitrks as emerging candidate genes involved in neuropsychiatric disorders. Trends Neurosci. 2011;34(3):143-153. doi:10.1016/j.tins.2011.01.001 21315458 PMC3051006

[67] Hatayama M, Katayama KI, Kawahara Y, . SLITRK1-mediated noradrenergic projection suppression in the neonatal prefrontal cortex. Commun Biol. 2022;5(1):935. doi:10.1038/s42003-022-03891-y 36085162 PMC9463131

[68] Duman-Scheel M. Netrin and DCC: axon guidance regulators at the intersection of nervous system development and cancer. Curr Drug Targets. 2009;10(7):602-610. doi:10.2174/138945009788680428 19601764 PMC2756184

[69] Boutsikou T, Giotaki M, Gourgiotis D, . Cord blood netrin-1 and -4 concentrations in term pregnancies with normal, restricted and increased fetal growth. J Matern Fetal Neonatal Med. 2014;27(18):1849-1853. doi:10.3109/14767058.2014.905530 24716747

