# Supplementary material for: Cord Blood Proteomic Profiles, Birth Weight, and Early Life Growth Trajectories
Source: JAMA Netw Open. 2024 May 14;7(5):e2411246. doi: 10.1001/jamanetworkopen.2024.11246 (PMC11094560; doi:10.1001/jamanetworkopen.2024.11246)
Supplement: Supplement 1. — eMethods. Covariables, Olink Proteomics, and Bonferroni Correction eTable 1. Main Anthropometric and Lifestyle Characteristics of the Participating Mother-Child Pairs Compared With Those Who Participated in the Follow-Up Examination Before February 2022 eTable 2. Main Anthropometric and Lifestyle Characteristics of the Participating Mother-Child Pairs Compared With Those of all Mother-Child Pairs Eligible for Follow-Up Between October 2014 and February 2022 eTable 3. Multiple Linear Regression Models Examining the Association of Cord Blood Proteins With Birth Weight or BWR in the Large Proteomics Analysis eTable 4. Numeric Data of the Associations Between Cord Blood Proteins and the Infant’s Birth Weight and BWR eTable 5. Sensitivity Analyses of Associations of Cord Blood Proteins With Infant Body Weight or BWR After Exclusion of Diabetes Gravidarum, Preeclampsia, or Gestational Hypertension, or After Additional Adjustment for Paternal Age at Delivery, Paternal Education, or All Other Proteins Statistically Significantly Associated With Birth Weight or BWR, or Stratified by Sex eTable 6. Sensitivity Analyses of Associations of Cord Blood Proteins With Infant Body Weight or BWR, With an Interaction Term Between Cord Blood Protein Levels and Sex eTable 7. Numeric Data of the Associations of Child Weight, BMI z Score, or Waist Circumference With Cord Blood Proteins eFigure 1. Participant Flowchart Depicting the Selection of Participants Enrolled in the ENVIRONAGE Birth Cohort for Arriving at the Final Study Sample Size eFigure 2. Scatterplot Showing the Correlation Between the BMI z Score at 4 to 6 Years Old and Birth Weight Normalized for Gestational Age eFigure 3. Pearson Correlation Matrices Between the Proteins Statistically Significant Associated With Birth Weight and/or BWR in the Large Proteomics Analysis eReferences. [file jamanetwopen-e2411246-s001.pdf]

Supplemental Online Content

Van Pee T, Martens DS, Alfano R, et al. Cord blood proteomic profiles, birth weight, and early life growth trajectories. *JAMA Netw Open*. 2024;7(5):e2411246. doi:10.1001/jamanetworkopen.2024.11246

eMethods. Covariables, Olink Proteomics, and Bonferroni Correction

eTable 1. Main Anthropometric and Lifestyle Characteristics of the Participating Mother-Child Pairs Compared With Those Who Participated in the Follow-Up Examination Before February 2022.

eTable 2. Main Anthropometric and Lifestyle Characteristics of the Participating Mother-Child Pairs Compared With Those of all Mother-Child Pairs Eligible for Follow-Up Between October 2014 and February 2022

eTable 3. Multiple Linear Regression Models Examining the Association of Cord Blood Proteins With Birth Weight or BWR in the Large Proteomics Analysis

eTable 4. Numeric Data of the Associations Between Cord Blood Proteins and the Infant's Birth Weight and BWR

eTable 5. Sensitivity Analyses of Associations of Cord Blood Proteins With Infant Body Weight or BWR After Exclusion of Diabetes Gravidarum, Preeclampsia, or Gestational Hypertension, or After Additional Adjustment for Paternal Age at Delivery, Paternal Education, or All Other Proteins Statistically Significantly Associated With Birth Weight or BWR, or Stratified by Sex

eTable 6. Sensitivity Analyses of Associations of Cord Blood Proteins With Infant Body Weight or BWR, With an Interaction Term Between Cord Blood Protein Levels and Sex

eTable 7. Numeric Data of the Associations of Child Weight, BMI z Score, or Waist Circumference With Cord Blood Proteins

eFigure 1. Participant Flowchart Depicting the Selection of Participants Enrolled in the ENVIRONAGE Birth Cohort for Arriving at the Final Study Sample Size

eFigure 2. Scatterplot Showing the Correlation Between the BMI z Score at 4 to 6 Years Old and Birth Weight Normalized for Gestational Age

eFigure 3. Pearson Correlation Matrices Between the Proteins Statistically Significant Associated With Birth Weight and/or BWR in the Large Proteomics Analysis

eReferences

This supplemental material has been provided by the authors to give readers additional information about their work.

© 2024 Van Pee T et al. *JAMA Network Open.*

Supplementary Material and methods

COVARIABLES

Parity was categorized as mothers having their first-, second-, or third or more child. Ethnicity was classified as European when two or more grandparents were of European descent. Maternal education was coded 'low' when the mother did not obtain a high school diploma, 'middle' when the mother obtained a high school diploma, and 'high' when the mother obtained a college or university degree. Smoking during pregnancy was self-reported and classified as 'no' when the mother reported she did not smoke during pregnancy.

OLINK PROTEOMICS

Pairs of oligonucleotide-labeled antibody probes against the same protein bind to their target, bringing the complementary oligonucleotides in close proximity and allowing for their hybridization. The addition of a DNA polymerase leads to the extension of the hybridized oligonucleotides, generating a unique protein identification "barcode". Next, library preparation adds sample identification indexes and the required nucleotides for Illumina sequencing. Prior to sequencing using the Illumina NovaSeq™ 6000, libraries go through a bead-based purification step and the quality is assessed using the Agilent 2100 Bioanalyzer (Agilent Technologies, Palo Alto, CA). The raw output data is quality controlled, normalized and converted into Normalized Protein eXpression (NPX) values (log_2). Three internal controls are spiked into every sample and are used to monitor the performance of the three main steps in the protocol: an incubation control, an extension control and an amplification control. In parallel with the samples, the protocol is performed on a set of external controls: two sample controls, three negative controls and three plate controls (PCs; used for between-plate normalization). Quality control is performed for each sample plate on both the samples (using the spiked internal controls) and the external controls. For all samples, the average counts may not fall below 500 counts and the

© 2024 Van Pee T et al. *JAMA Network Open.*

incubation/amplification controls may not deviate from the median more than 0.3 NPX. Exceeding any of these criteria will result in a QC warning. For the PCs and negative controls, the median of the triplicates may not exceed more than 5 standard deviations (SD) and 3 SDs, respectively, from predefined values for more than 10% of the assays. For the negative controls, only positive deviations are considered. Assays not fulfilling these criteria will receive a QC warning. If the number of samples with a QC warning exceeds 1/6 of the samples on a plate and the median absolute deviation (MAD) for incubation or amplification control across all samples exceeds 0.3, the protocol is considered failed. All assay validation data are available on manufacturer's website (www.olink.com).

BONFERRONI CORRECTION

In this study, the association between 288 cord blood proteins and multiple (birth) weight-related outcomes was investigated. Since this increases the risk of a type I error[1], we used Bonferroni correction adjusted p-values. The Bonferroni correction method formula is α/n in which α is the original alpha level (*e.g.,* 0.05) and n is the number of tests being performed (here 368).

© 2024 Van Pee T et al. *JAMA Network Open.*

eTable 1: Main anthropometric and lifestyle characteristics of the participating mother-child pairs (n = 288) compared to the characteristics of all mother-child pairs that participated in the follow-up examination before February 2022.

Characteristic	Random subset (n = 288)	FU participants before February 2022 (n = 588)	p-value
**Mother**			
**Age at delivery** (years)	30.4±4.1	30.1±4.3	0.38
**Pre-pregnancy BMI** (kg/m^2)	24.2±4.3	24.3±4.4	0.64
**Parity**			
First child	151 (52.4%)	318 (54.1%)	0.89
Second child	108 (37.5%)	212 (36.1%)	
Third child	29 (10.1%)	58 (9.8%)	
**Smoking during pregnancy**			
No	265 (92.0%)	532 (90.5%)	0.22
**Maternal education**			
Low	18 (6.3%)	37 (6.3%)	0.64
Middle	79 (27.4%)	168 (28.6%)	
High	191 (66.3%)	383 (65.1%)	
**Child**			
**Sex**			
Boy	125 (43.4%)	280 (47.6%)	0.24
**Gestational age** (days)	277.2±11.6	276.7±11.3	0.54
**Birth weight** (grams)	3389.6±492.9	3396.7±506.4	0.85
**BWR**	1.0±0.1	1.0±0.1	0.71
**European**	274 (95.1%)	557 (94.7%)	0.54
**Rapid growth at 12 months**			
Yes	91 (31.6%)	189 (32.1%)	0.52
**Age at FU** (years)	4.6±0.4	4.6±0.4	0.73
**Weight at FU** (kg)	18.6±2.6	18.8±2.5	0.30
**BMI z-score at FU**	0.5±0.8	0.5±0.8	0.82
**Waist circumference at FU** (cm)	53.0±3.6	53.3±3.8	0.28
**Child overweight** (including obesity)			
Yes	34 (11.8%)	82 (13.9%)	0.29

Continuous covariables are expressed as mean ± SD and categorical covariables as total number (n) and percentage (%). The p-value indicates whether the covariables differ between the random subset and the FU participants before February 2022. Parity was categorized as mothers having their first-, second-, or third or more child. Maternal educational level was coded "low" if the participant did not obtain a high school diploma, "middle" if the participant obtained a high school diploma, and "high" if the participant obtained a college or university degree. Ethnicity was based on the native country of the newborn's grandparents and described as European when two or more grandparents were European or non-European when at least three grandparents were of non-European origin. BMI: body mass index; BWR: birth weight ratio; FU: follow-up examination.

© 2024 Van Pee T et al. *JAMA Network Open.*

eTable 2: Main anthropometric and lifestyle characteristics of the participating mother-child pairs (n = 288) compared to those of all mother-child pairs eligible for FU between October 2014 and February 2022.

Characteristic	Random subset (n = 288)	Eligible for FU between October 2014 and February 2022 (n = 1325)*	p-value
**Mother**			
**Age at delivery** (years)	30.4±4.1	29.4±4.5	<0.001
**Pre-pregnancy BMI** (kg/m^2)	24.2±4.3	24.5±4.7	0.30
**Parity**			
First child	151 (52.4%)	701 (53.1%)	0.66
Second child	108 (37.5%)	465 (35.3%)	
Third child	29 (10.1%)	153 (11.6%)	
**Smoking during pregnancy**			
No	265 (92.0%)	1189 (90.1%)	0.33
**Maternal education**			
Low	18 (6.3%)	148 (11.2%)	<0.001
Middle	79 (27.4%)	456 (34.6%)	
High	191 (66.3%)	715 (54.2%)	
**Child**			
**Sex**			
Boy	125 (43.4%)	670 (50.6%)	<0.001
**Gestational age** (days)	277.2±11.6	277.0±11.0	0.75
**Birth weight** (grams)	3389.6±492.9	3396.2±492.2	0.84
**BWR**	1.0±0.1	1.0±0.1	0.88
**European**	274 (95.1%)	1151 (87.3%)	<0.001

Continuous covariables are expressed as mean ± SD and categorical covariables as total number (n) and percentage (%). The p-value indicates whether the covariables differ between the random subset and all of the ENVIR*ON*AGE participants before February 2022. Parity was categorized as mothers having their first-, second-, or third or more child. Maternal educational level was coded "low" if the participant did not obtain a high school diploma, "middle" if the participant obtained a high school diploma, and "high" if the participant obtained a college or university degree. Ethnicity was based on the native country of the newborn's grandparents and described as European when two or more grandparents were European or non-European when at least three grandparents were of non-European origin. BMI: body mass index; BWR: birth weight ratio; FU: follow-up. *Data available for 1319 mother-child pairs.

© 2024 Van Pee T et al. *JAMA Network Open.*

eTable 3: Results of the multiple linear regression models examining the association between cord blood proteins and birth weight or BWR in the large proteomics analysis.

Cord blood protein	Protein identifier	Birth weight		BWR	
		Difference (gram)	p_{adj}-value	Difference	p_{adj}-value
A1BG	P04217	42.75	1.00	0.02	1.00
ABO	P16442	-2.15	1.00	-0.002	1.00
ACE	P12821	-43.21	1.00	-0.01	1.00
ACHE	P22303	14.49	1.00	-0.001	1.00
ACP1	P24666	-50.14	1.00	-0.02	1.00
ACRV1	P26436	50.76	1.00	0.01	1.00
ACYP1	P07311	-30.98	1.00	-0.01	1.00
ADAM12	O43184	129.23	1.00	0.03	1.00
ADAMTS1	Q9UHI8	-26.48	1.00	-0.006	1.00
ADAMTS4	O75173	1.30	1.00	0.005	1.00
ADD1	P35611	-20.84	1.00	-0.007	1.00
ADGRD1	Q6QNK2	-7.86	1.00	-0.009	1.00
ADH1B	P00325	35.04	1.00	0.01	1.00
ADIPOQ	Q15848	10.43	1.00	0.004	1.00
AFAP1	Q8N556	9.53	1.00	0.002	1.00
AFM	P43652	341.16	0.003	0.09	0.07
AGT	P01019	21.10	1.00	0.01	1.00
AHSG	P02765	33.35	1.00	0.007	1.00
AKAP12	Q02952	-127.24	1.00	-0.05	1.00
AKR7L	Q8NHP1	-69.91	1.00	-0.02	1.00
ALPI	P09923	-32.36	1.00	-0.006	1.00
AMOT	Q4VCS5	41.15	1.00	0.01	1.00
AMY1A_AMY1B_AMY1C	P0DUB6_P0DTE7_P0DTE8	-25.16	1.00	-0.005	1.00
ANKMY2	Q8IV38	9.45	1.00	0.003	1.00
ANXA1	P04083	-45.31	1.00	-0.01	1.00
APCS	P02743	-2.70	1.00	0.006	1.00
APOA1	P02647	39.13	1.00	0.009	1.00
APOA2	P02652	25.02	1.00	0.007	1.00
APOA4	P06727	-117.12	1.00	-0.04	1.00
APOB	P04114	-123.35	1.00	-0.04	1.00
APOC1	P02654	26.56	1.00	0.009	1.00
APOD	P05090	-51.99	1.00	-0.02	1.00
APOE	P02649	2.32	1.00	-0.002	1.00
APOF	Q13790	-65.99	1.00	-0.01	1.00
APOL1	O14791	5.64	1.00	0.008	1.00
APPL2	Q8NEU8	-0.53	1.00	0.000	1.00
ARHGAP45	Q92619	2.04	1.00	0.001	1.00
ASGR2	P07307	140.94	1.00	0.04	1.00
ATRN	O75882-2	-15.42	1.00	-0.006	1.00
B2M	P61769	50.75	1.00	0.01	1.00
BABAM1	Q9NWV8	-45.47	1.00	-0.02	1.00
BAG4	O95429	5.74	1.00	0.001	1.00
BCHE	P06276	55.76	1.00	0.02	1.00
BCL2L15	Q5TBC7	4.77	1.00	0.001	1.00
BLNK	Q8WV28	-10.24	1.00	-0.002	1.00
BMP10	O95393	91.29	1.00	0.03	1.00
BMPER	Q8N8U9	104.00	1.00	0.03	1.00
BNIP3L	O60238	22.94	1.00	0.006	1.00
BTD	P43251	108.54	1.00	0.03	1.00
C1QL2	Q7Z5L3	53.83	1.00	0.01	1.00
C1QTNF5	Q9BXJ0	-23.66	1.00	-0.008	1.00
C1QTNF9	P0C862	2.95	1.00	0.003	1.00
C1R	P00736	42.35	1.00	0.02	1.00
C1RL	Q9NZP8	128.97	1.00	0.04	1.00
C1S	P09871	45.79	1.00	0.02	1.00
C3	P01024	63.71	1.00	0.02	1.00
C5	P01031	34.24	1.00	0.01	1.00
C7	P10643	-73.61	1.00	-0.02	1.00
C8B	P07358	72.00	1.00	0.03	1.00
C9	P02748	-9.19	1.00	0.002	1.00
CA8	P35219	-55.24	1.00	-0.01	1.00
CACYBP	Q9HB71	5.45	1.00	0.002	1.00
CASP9	P55211	-2.54	1.00	-0.001	1.00
CAT	P04040	-35.10	1.00	-0.01	1.00
CCNE1	P24864	23.65	1.00	0.01	1.00
CD226	Q15762	-3.26	1.00	-0.004	1.00

© 2024 Van Pee T et al. *JAMA Network Open.*

CD300A	Q9UGN4	-13.62	1.00	-0.004	1.00
CD36	P16671	-34.66	1.00	-0.01	1.00
CD3G	P09693	-6.21	1.00	0.002	1.00
CD5L	O43866	79.76	1.00	0.02	1.00
CD7	P09564	-4.37	1.00	-0.008	1.00
CD72	P21854	48.12	1.00	0.02	1.00
CEBPA	P49715	23.63	1.00	0.009	1.00
CELSR2	Q9HCU4	-237.52	0.01	-0.06	0.20
CEMIP2	Q9UHN6	13.73	1.00	0.02	1.00
CFB	P00751	79.28	1.00	0.03	1.00
CFD	P00746	162.00	1.00	0.05	1.00
CFH	P08603	130.15	1.00	0.04	1.00
CFHR2	P36980	48.43	1.00	0.02	1.00
CFHR4	Q92496	200.70	1.00	0.06	1.00
CFHR5	Q9BXR6	-42.30	1.00	-0.01	1.00
CFI	P05156	467.28	0.61	0.15	0.62
CFP	P27918	67.95	1.00	0.01	1.00
CGB3_CGB5_CGB8	P0DN86	-5.55	1.00	-0.005	1.00
CHAD	O15335	-209.13	1.00	-0.07	1.00
CLEC12A	Q5QGZ9	14.51	1.00	0.001	1.00
CLEC3B	P05452	77.47	1.00	0.03	1.00
CLU	P10909	56.60	1.00	0.02	1.00
COL5A1	P20908	70.23	1.00	0.01	1.00
CPA4	Q9UI42	7.42	1.00	-0.003	1.00
CPB2	Q96IY4	117.61	1.00	0.04	1.00
CPOX	P36551	118.65	1.00	0.03	1.00
CR1	P17927	-37.21	1.00	-0.009	1.00
CRELD1	Q96HD1	-20.28	1.00	-0.006	1.00
CRISP3	P54108	-117.77	1.00	-0.02	1.00
CSF1R	P07333	0.56	1.00	0.000	1.00
CSF2RB	P32927	-37.40	1.00	-0.003	1.00
CSF3R	Q99062	55.77	1.00	0.03	1.00
CSH1	P0DML2	3.17	1.00	0.0001	1.00
CSNK1D	P48730	-1.97	1.00	-0.001	1.00
CST1	P01037	-6.72	1.00	-0.003	1.00
CTBS	Q01459	73.39	1.00	0.02	1.00
CTSE	P14091	-25.26	1.00	-0.007	1.00
DAAM1	Q9Y4D1	-11.33	1.00	-0.002	1.00
DAND5	Q8N907	8.24	1.00	0.001	1.00
DAPK2	Q9UIK4	-11.35	1.00	-0.004	1.00
DBH	P09172	4.79	1.00	0.006	1.00
DBN1	Q16643	3.43	1.00	-0.0002	1.00
DCTD	P32321	-0.14	1.00	0.0003	1.00
DDI2	Q5TDH0	-37.54	1.00	-0.01	1.00
DDX39A	O00148	45.69	1.00	0.02	1.00
DDX4	Q9NQI0	-43.47	1.00	-0.02	1.00
DEFB103A_DEFB103B	P81534	11.49	1.00	0.001	1.00
DENR	O43583	-13.95	1.00	-0.006	1.00
DGKA	P23743	-10.57	1.00	-0.004	1.00
DIPK2B	Q9H7Y0	-9.06	1.00	-0.001	1.00
DNAJB2	P25686	-27.60	1.00	-0.01	1.00
DNAJB6	O75190	-8.50	1.00	-0.004	1.00
DTD1	Q8TEA8	0.20	1.00	0.0001	1.00
ECM1	Q16610	80.50	1.00	0.02	1.00
EDNRB	P24530	-4.17	1.00	-0.006	1.00
EIF4E	P06730	-7.82	1.00	-0.003	1.00
EP300	Q09472	38.81	1.00	0.008	1.00
EPHA4	P54764	-342.78	<0.001	-0.11	<0.001
ERMAP	Q96PL5	34.86	1.00	0.01	1.00
ERP29	P30040	6.87	1.00	0.002	1.00
ESR1	P03372	30.95	1.00	0.005	1.00
EVI5	O60447	-1.50	1.00	-0.0002	1.00
F10	P00742	188.44	1.00	0.07	1.00
F11	P03951	56.94	1.00	0.02	1.00
F12	P00748	76.69	1.00	0.03	1.00
F13B	P05160	70.87	1.00	0.02	1.00
F2	P00734	180.69	1.00	0.06	1.00
FCN1	O00602	-6.07	1.00	0.001	1.00
FGA	P02671	30.67	1.00	0.01	1.00
FGF12	P61328	-90.08	1.00	-0.03	1.00
FGF16	O43320	-54.87	1.00	-0.02	1.00
FGF20	Q9NP95	-86.21	1.00	-0.03	1.00

© 2024 Van Pee T et al. *JAMA Network Open.*

FGF3	P11487	-66.36	1.00	-0.01	1.00
FGF6	P10767	-27.31	1.00	-0.02	1.00
FGFR4	P22455	16.02	1.00	0.003	1.00
FGL1	Q08830	-39.05	1.00	-0.007	1.00
FN1	P02751	26.63	1.00	0.009	1.00
FOLH1	Q04609	-97.34	1.00	-0.02	1.00
FOXJ3	Q9UPW0	6.30	1.00	0.002	1.00
FUOM	A2VDF0	-57.26	1.00	-0.01	1.00
GAD2	Q05329	-58.37	1.00	-0.03	1.00
GAPDH	P04406	-20.77	1.00	-0.005	1.00
GC	P02774	148.28	1.00	0.05	0.63
GCHFR	P30047	-35.93	1.00	-0.01	1.00
GHR	P10912	5.34	1.00	0.002	1.00
GIMAP7	Q8NHV1	-52.80	1.00	-0.02	1.00
GIPR	P48546	-55.17	1.00	-0.02	1.00
GIT1	Q9Y2X7	-5.30	1.00	-0.002	1.00
GLA	P06280	-23.45	1.00	-0.005	1.00
GLI2	P10070	-55.69	1.00	-0.02	1.00
GLRX5	Q86SX6	-2.13	1.00	-0.0004	1.00
GMPR2	Q9P2T1	-18.82	1.00	-0.007	1.00
GNPDA2	Q8TDQ7	-52.40	1.00	-0.02	1.00
GP5	P40197	-33.63	1.00	-0.01	1.00
GPI	P06744	-12.86	1.00	-0.005	1.00
GSN	P06396	-102.04	1.00	-0.04	1.00
GSR	P00390	-107.57	1.00	-0.04	1.00
HDAC8	Q9BY41	73.23	1.00	0.009	1.00
HEG1	Q9ULI3	-69.99	1.00	-0.01	1.00
HGFAC	Q04756	20.91	1.00	0.02	1.00
HIF1A	Q16665	-28.35	1.00	-0.005	1.00
HMCN2	Q8NDA2	84.83	1.00	0.02	1.00
HRG	P04196	11.34	1.00	0.005	1.00
HS6ST2	Q96MM7	-134.40	1.00	-0.04	1.00
IDO1	P14902	-7.15	1.00	-0.003	1.00
IGFL4	Q6B9Z1	61.80	1.00	0.02	1.00
IGLC2	P0DOY2	-45.52	1.00	-0.02	1.00
IL12RB2	Q99665	-19.83	1.00	-0.02	1.00
IL20RB	Q6UXL0	63.40	1.00	0.01	1.00
IL21R	Q9HBE5	-104.29	1.00	-0.04	1.00
IL31	Q6EBC2	-114.76	1.00	-0.03	1.00
IL31RA	Q8NI17	-39.92	1.00	-0.02	1.00
IL36A	Q9UHA7	7.31	1.00	0.002	1.00
IL36G	Q9NZH8	0.37	1.00	-0.002	1.00
INHBB	P09529	-70.18	1.00	-0.01	1.00
INSR	P06213	-271.15	0.55	-0.07	1.00
ITGA2	P17301	-205.44	1.00	-0.07	0.81
ITGAL	P20701	-42.62	1.00	-0.01	1.00
ITIH1	P19827	108.71	1.00	0.03	1.00
ITIH4	Q14624	36.48	1.00	0.02	1.00
JAM3	Q9BX67	-47.58	1.00	-0.02	1.00
KDM3A	Q9Y4C1	-32.35	1.00	-0.02	1.00
KLK7	P49862	40.48	1.00	0.004	1.00
KLKB1	P03952	102.90	1.00	0.04	1.00
KLRF1	Q9NZS2	-49.11	1.00	-0.02	1.00
LATS1	O95835	5.35	1.00	0.002	1.00
LCAT	P04180	129.57	1.00	0.04	1.00
LEG1	Q6P5S2	-30.48	1.00	-0.006	1.00
LGALS3BP	Q08380	77.68	1.00	0.02	1.00
LMOD1	P29536	-13.00	1.00	-0.004	1.00
LPA	P08519	25.99	1.00	0.007	1.00
LRG1	P02750	-23.42	1.00	0.007	1.00
LRIG3	Q6UXM1	-53.86	1.00	-0.025	1.00
LYVE1	Q9Y5Y7	-42.34	1.00	-0.014	1.00
LZTFL1	Q9NQ48	-8.85	1.00	-0.004	1.00
MARS1	P56192	-1.80	1.00	-0.0003	1.00
MBL2	P11226	31.60	1.00	0.01	1.00
MCEMP1	Q8IX19	-14.70	1.00	-0.003	1.00
MDH1	P40925	-45.81	1.00	-0.02	1.00
MENT	Q9BUN1	76.34	1.00	0.04	1.00
MFAP4	P55083	-29.47	1.00	-0.01	1.00
MKI67	P46013	31.43	1.00	0.01	1.00
MOCS2	O96007	-0.20	1.00	0.0003	1.00
MRC1	P22897	-15.48	1.00	0.007	1.00

© 2024 Van Pee T et al. *JAMA Network Open.*

MRPS16	Q9Y3D3	-41.63	1.00	-0.02	1.00
MST1	P26927	18.82	1.00	0.008	1.00
MTDH	Q86UE4	19.01	1.00	0.006	1.00
MXRA8	Q9BRK3	2.88	1.00	-0.01	1.00
MYOM3	Q5VTT5	-46.35	1.00	-0.01	1.00
NAGA	P17050	11.81	1.00	0.001	1.00
NAGPA	Q9UK23	17.99	1.00	0.001	1.00
NDUFA5	Q16718	-49.45	1.00	-0.02	1.00
NECTIN1	Q15223	-36.46	1.00	-0.008	1.00
NEDD4L	Q96PU5	-4.97	1.00	-0.005	1.00
NEDD9	Q14511	-25.56	1.00	-0.01	1.00
NEXN	Q0ZGT2	16.31	1.00	0.006	1.00
NFAT5	O94916	-7.94	1.00	-0.003	1.00
NHLRC3	Q5JS37	0.71	1.00	0.00001	1.00
NME1	P15531	31.12	1.00	0.01	1.00
NPHS1	O60500	-93.36	1.00	-0.03	1.00
NPHS2	Q9NP85	61.76	1.00	0.01	1.00
NRGN	Q92686	-0.84	1.00	0.0004	1.00
NUMB	P49757	-9.81	1.00	-0.004	1.00
NXPH3	O95157	-116.59	1.00	-0.04	1.00
OLFM4	Q6UX06	-25.58	1.00	-0.003	1.00
ORM1	P02763	-9.84	1.00	0.008	1.00
PALLD	Q8WX93	50.79	1.00	0.01	1.00
PAXX	Q9BUH6	-32.69	1.00	-0.01	1.00
PCBD1	P61457	-26.76	1.00	-0.008	1.00
PDE5A	O76074	0.12	1.00	0.0002	1.00
PDIA3	P30101	19.35	1.00	0.008	1.00
PDZK1	Q5T2W1	2.80	1.00	0.004	1.00
PENK	P01210	-157.02	1.00	-0.05	1.00
PEPD	P12955	-106.55	1.00	-0.03	1.00
PER3	P56645	2.06	1.00	0.001	1.00
PF4	P02776	-4.21	1.00	-0.001	1.00
PGA4	P0DJD7	-53.49	1.00	-0.006	1.00
PGLYRP2	Q96PD5	124.49	1.00	0.04	1.00
PGR	P06401	120.08	1.00	0.03	1.00
PHYKPL	Q8IUZ5	-13.57	1.00	-0.005	1.00
PI16	Q6UXB8	150.95	1.00	0.03	1.00
PIKFYVE	Q9Y2I7	-1.76	1.00	-0.002	1.00
PINLYP	A6NC86	-32.12	1.00	-0.01	1.00
PLCB1	Q9NQ66	154.94	1.00	0.04	1.00
PLG	P00747	108.50	1.00	0.03	1.00
PNLIP	P16233	-20.45	1.00	-0.006	1.00
POF1B	Q8WVV4	-64.23	1.00	-0.02	1.00
POLR2A	P24928	78.51	1.00	0.03	1.00
PON1	P27169	19.24	1.00	0.006	1.00
PPBP	P02775	-5.59	1.00	-0.001	1.00
PPL	O60437	-74.98	1.00	-0.02	1.00
PPM1B	O75688	54.20	1.00	0.01	1.00
PRDX2	P32119	-42.39	1.00	-0.01	1.00
PRKG1	Q13976	3.14	1.00	0.001	1.00
PROS1	P07225	71.52	1.00	0.03	1.00
PRR4	Q16378	-50.24	1.00	-0.01	1.00
PRR5	P85299	7.44	1.00	-0.004	1.00
PRSS22	Q9GZN4	-89.11	1.00	-0.02	1.00
PSMG4	Q5JS54	-31.84	1.00	-0.01	1.00
PSTPIP2	Q9H939	2.73	1.00	0.001	1.00
PTGES2	Q9H7Z7	-1.59	1.00	-0.001	1.00
PTP4A3	O75365	-31.96	1.00	-0.01	1.00
PTPN9	P43378	-59.06	1.00	-0.03	1.00
PTRHD1	Q6GMV3	-17.43	1.00	-0.006	1.00
PZP	P20742	18.39	1.00	0.006	1.00
QSOX1	O00391	75.22	1.00	0.02	1.00
RABEP1	Q15276	-17.36	1.00	-0.006	1.00
RALB	P11234	41.28	1.00	0.01	1.00
RAP1A	P62834	-34.09	1.00	-0.02	1.00
RBPMS	Q93062	-14.55	1.00	-0.004	1.00
RELB	Q01201	-94.63	1.00	-0.02	1.00
REPS1	Q96D71	-6.12	1.00	-0.004	1.00
RICTOR	Q6R327	-60.82	1.00	-0.02	1.00
RIDA	P52758	-24.60	1.00	-0.008	1.00
RLN2	P04090	-7.49	1.00	-0.005	1.00
RNASE1	P07998	82.66	1.00	0.02	1.00

© 2024 Van Pee T et al. *JAMA Network Open.*

RNASE4	P34096	21.45	1.00	0.009	1.00
RNASE6	Q93091	89.30	1.00	0.03	1.00
RNF168	Q8IYW5	51.03	1.00	0.01	1.00
RNF31	Q96EP0	-6.95	1.00	-0.01	1.00
RPA2	P15927	-22.37	1.00	-0.007	1.00
S100A13	Q99584	-80.47	1.00	-0.03	1.00
SAA4	P35542	158.69	1.00	0.05	1.00
SCGB3A1	Q96QR1	-89.01	1.00	-0.03	1.00
SCRIB	Q14160	2.39	1.00	0.001	1.00
SDK2	Q58EX2	-168.30	1.00	-0.06	1.00
SELENOP	P49908	81.31	1.00	0.03	1.00
SELL	P14151	87.03	1.00	0.03	1.00
SEMA3G	Q9NS98	-10.90	1.00	-0.001	1.00
SEMA6C	Q9H3T2	-127.55	1.00	-0.05	1.00
SERPINA1	P01009	-127.98	1.00	-0.02	1.00
SERPINA3	P01011	-25.01	1.00	0.003	1.00
SERPINA4	P29622	140.00	1.00	0.04	1.00
SERPINA5	P05154	89.87	1.00	0.03	1.00
SERPINA6	P08185	31.57	1.00	0.006	1.00
SERPINA7	P05543	156.43	1.00	0.05	1.00
SERPINC1	P01008	193.98	1.00	0.07	1.00
SERPIND1	P05546	150.92	1.00	0.05	1.00
SERPINF1	P36955	245.33	1.00	0.05	1.00
SERPINF2	P08697	146.25	1.00	0.05	1.00
SERPING1	P05155	-11.73	1.00	0.004	1.00
SERPINI1	Q99574	-13.05	1.00	0.002	1.00
SFRP4	Q6FHJ7	242.60	0.001	0.067	0.01
SHBG	P04278	-139.57	1.00	-0.03	1.00
SHH	Q15465	-1.69	1.00	0.001	1.00
SIRT1	Q96EB6	19.12	1.00	0.007	1.00
SLC9A3R1	O14745	2.17	1.00	0.001	1.00
SLITRK1	Q96PX8	-366.32	<0.001	-0.11	<0.001
SMPD3	Q9NY59	-52.11	1.00	-0.03	1.00
SNCA	P37840	-25.64	1.00	-0.008	1.00
SNX15	Q9NRS6	-19.72	1.00	-0.007	1.00
SOD3	P08294	-16.53	1.00	-0.003	1.00
SPART	Q8N0X7	-1.39	1.00	-0.001	1.00
SPINK2	P20155	-26.16	1.00	-0.01	1.00
SPRED2	Q7Z698	-18.74	1.00	-0.006	1.00
SSBP1	Q04837	-27.45	1.00	-0.009	1.00
ST13	P50502	-35.54	1.00	-0.01	1.00
ST8SIA1	Q92185	67.84	1.00	0.02	1.00
STAT2	P52630	0.70	1.00	-0.001	1.00
STX5	Q13190	-15.09	1.00	-0.008	1.00
STX7	O15400	-30.46	1.00	-0.01	1.00
SUMF1	Q8NBK3	-10.62	1.00	-0.006	1.00
SUSD5	O60279	-280.86	0.39	-0.09	0.20
SYAP1	Q96A49	-20.68	1.00	-0.008	1.00
TBCA	O75347	-14.77	1.00	-0.005	1.00
TCN1	P20061	-208.75	0.01	-0.06	0.08
TERF1	P54274	-6.49	1.00	-0.001	1.00
TF	P02787	36.15	1.00	0.02	1.00
TGFBR1	P36897	-272.99	0.45	-0.09	0.22
TGOLN2	O43493	-115.28	1.00	-0.04	1.00
THSD1	Q9NS62	119.27	1.00	0.04	1.00
TLR1	Q15399	-9.13	1.00	-0.006	1.00
TLR4	O00206	51.58	1.00	0.02	1.00
TNFAIP8L2	Q6P589	-2.88	1.00	-0.002	1.00
TNFRSF17	Q02223	-37.63	1.00	-0.02	1.00
TOP2B	P41273	-2.29	1.00	-0.001	1.00
TP53BP1	Q02880	5.02	1.00	0.002	1.00
TP53I3	Q12888	-22.77	1.00	-0.009	1.00
TPD52L2	Q53FA7	0.22	1.00	0.0002	1.00
TPSG1	O43399	107.75	1.00	0.030	1.00
TRAF3	Q9NRR2	-34.08	1.00	-0.01	1.00
TREML1	Q13114-2	-11.58	1.00	-0.004	1.00
TSC1	Q86YW5	-50.47	1.00	-0.02	1.00
TSPYL1	Q92574	-11.28	1.00	-0.007	1.00
TTR	Q9H0U9	173.59	1.00	0.05	1.00
TXN	P02766	-12.57	1.00	-0.007	1.00
TYRP1	P10599	29.11	1.00	0.007	1.00
UBE2Z	P17643	-6.17	1.00	-0.003	1.00

© 2024 Van Pee T et al. *JAMA Network Open.*

UBXN1	Q9H832	-19.85	1.00	-0.007	1.00
UNC5D	Q04323	-209.27	0.001	-0.07	0.001
UROD	Q6UXZ4	-16.93	1.00	-0.006	1.00
VAMP8	P06132	2.70	1.00	0.0004	1.00
VASP	Q9BV40	8.85	1.00	0.003	1.00
VEGFB	P50552	23.74	1.00	-0.002	1.00
VNN1	P49765	-8.50	1.00	-0.003	1.00
VTI1A	O95497	-21.89	1.00	-0.007	1.00
WASL	Q96AJ9	-31.33	1.00	-0.01	1.00
XIAP	O00401	7.38	1.00	0.002	1.00
YWHAQ	P98170	-6.34	1.00	-0.003	1.00
YY1	P27348	-46.13	1.00	-0.2	1.00
ZBP1	P25490	-70.92	1.00	-0.02	1.00
ZNF174	Q9H171	-17.43	1.00	-0.004	1.00

Table of 368 proteins with protein identifiers showing the difference in infant's birth weight (gram) and BWR with false discovery rate adjusted p-value per doubling in cord blood protein levels. Multiple linear regression models were adjusted for sex, gestational age, maternal pre-pregnancy BMI, maternal age at delivery, date of delivery, ethnicity, parity, smoking during pregnancy, and maternal education. BWR: birth weight ratio.

© 2024 Van Pee T et al. *JAMA Network Open.*

eTable 4: Numeric data of the associations between cord blood proteins and the infant's birth weight and BWR. Only cord blood proteins that remained statistically significant after multiple testing correction with Bonferroni are shown.

Cord blood protein	Birth weight				BWR			
	Difference (grams)	95% CI	p-value	p_{adj}-value	Difference	95% CI	p-value	p_{adj}-value
AFM	341.16	192.76 to 489.50	<0.001	0.003				
CELSR2	-237.52	-131.89 to -343.15	<0.001	0.005				
EPHA4	-342.78	-222.47 to -463.10	<0.001	<0.001	-0.11	-0.07 to -0.14	<0.001	<0.001
SFRP4	242.60	142.77 to 342.43	<0.001	0.001	0.07	0.04 to 0.10	<0.001	0.01
SLITRK1	-366.32	-255.97 to -476.66	<0.001	<0.001	-0.11	-0.08 to -0.15	<0.001	<0.001
TCN1	-208.75	-112.26 to -305.23	<0.001	0.01				
UNC5D	-209.27	-123.40 to -295.14	<0.001	<0.001	-0.07	-0.04 to -0.09	<0.001	<0.001

Results are expressed as difference with 95% CI in birth weight or BWR per doubling in cord blood protein levels. Multiple linear regression models were adjusted for sex, gestational age, maternal pre-pregnancy BMI, maternal age at delivery, month and year of delivery, ethnicity, parity, smoking during pregnancy, and maternal education. BWR: birth weight ratio; CI: confidence interval.

© 2024 Van Pee T et al. *JAMA Network Open.*

eTable 5: Sensitivity analyses of associations between cord blood proteins and the infant's body weight or BWR after exclusion of diabetes gravidarum (n = 8), preeclampsia (n = 4), or gestational hypertension (n = 10), or after additional adjustment for paternal age at delivery, paternal education, or all other proteins statistically significantly associated with birth weight or BWR, or stratified by sex. In addition, a sensitivity analysis was performed in which the linear regression models of BWR were not adjusted for sex, gestational age, and parity.

	**Cord blood protein**	**Birth weight**		**BWR**	
		Difference (gram)	95% CI	Difference	95% CI
**Exclusion of diabetes gravidarum (n = 280)**	AFM	338.22	187.75 to 488.67	0.09	0.04 to 0.14
	CELSR2	-248.92	-141.77 to -355.98	-0.06	-0.03 to -0.10
	EPHA4	-347.10	-225.69 to -468.50	-0.11	-0.07 to -0.15
	SFRP4	242.05	140.26 to 343.78	0.07	0.03 to 0.10
	SLITRK1	-369.25	-257.56 to -480.93	-0.11	-0.08 to -0.15
	TCN1	-210.36	-111.72 to -308.95	-0.06	-0.03 to -0.09
	UNC5D	-206.38	-119.45 to -293.22	-0.07	-0.04 to -0.09
**Exclusion of preeclampsia during pregnancy (n = 284)**	AFM	348.12	198.74 to 497.56	0.09	0.05 to 0.14
	CELSR2	-231.97	-124.65 to -339.05	-0.06	-0.02 to -0.09
	EPHA4	-338.54	-216.06 to -461.01	-0.10	-0.07 to -0.14
	SFRP4	241.53	141.04 to 341.95	0.07	0.03 to 0.10
	SLITRK1	-361.57	-249.14 to -473.94	-0.11	-0.08 to -0.15
	TCN1	-209.31	-111.57 to -306.98	-0.06	-0.03 to -0.09
	UNC5D	-205.68	-118.79 to -292.39	-0.07	-0.04 to -0.09
**Exclusion of gestational hypertension (n = 278)**	AFM	346.37	196.28 to 496.27	0.09	0.04 to 0.14
	CELSR2	-229.44	-119.84 to -339.06	-0.06	-0.02 to -0.09
	EPHA4	-327.11	-204.88 to -449.35	-0.10	-0.06 to -0.14
	SFRP4	245.68	145.29 to 345.91	0.07	0.04 to 0.10
	SLITRK1	-349.72	-237.72 to -461.72	-0.11	-0.07 to -0.14
	TCN1	-200.51	-100.52 to -300.56	-0.06	-0.02 to -0.09
	UNC5D	-194.24	-106.95 to -281.42	-0.06	-0.03 to -0.09
**Additionally adjusted for paternal age at delivery (n = 246)**	AFM	350.07	181.85 to 518.19	0.10	0.05 to 0.15
	CELSR2	-252.11	-134.62 to -369.62	-0.06	-0.03 to -0.10
	EPHA4	-342.04	-209.82 to -474.12	-0.10	-0.06 to -0.15
	SFRP4	267.61	156.89 to 378.29	0.07	0.04 to 0.11
	SLITRK1	-375.92	-256.66 to -495.06	-0.12	-0.08 to -0.15
	TCN1	-245.18	-138.38 to -351.73	-0.07	-0.03 to -0.10
	UNC5D	-208.73	-115.15 to -302.27	-0.10	-0.04 to -0.15
**Additionally adjusted for paternal education (n = 181)**	AFM	355.38	185.50 to 525.09	0.10	0.05 to 0.15
	CELSR2	-262.61	-145.34 to -379.95	-0.07	-0.03 to -0.10
	EPHA4	-349.01	-215.49 to -482.49	-0.11	-0.06 to -0.15
	SFRP4	262.77	150.75 to 374.63	0.07	0.04 to 0.11
	SLITRK1	-377.65	-257.06 to -498.13	-0.12	-0.08 to -0.15
	TCN1	-257.06	-150.20 to -363.75	-0.07	-0.04 to -0.10
	UNC5D	-211.42	-116.35 to -306.46	-0.07	-0.04 to -0.10
**Additionally adjusted for the other statistically significant proteins (n = 288)**	AFM	358.22	207.19 to 509.30		
	CELSR2	-119.34	-3.14 to -235.40		
	EPHA4	-245.48	-32.53 to -458.27	-0.07	-0.01 to -0.14
	SFRP4	243.77	142.18 to 345.16	0.09	0.05 to 0.12
	SLITRK1	-299.97	-93.40 to -506.32	-0.11	-0.04 to -0.17
	TCN1	-97.84	-201.39 to 5.72		
	UNC5D	-147.35	-300.83 to 6.27	-0.03	-0.08 to 0.02
**Stratified by boys (n = 125)**	AFM	304.61	73.32 to 535.91		
	CELSR2	-246.83	-65.67 to -427.87		
	EPHA4	-317.45	-120.04 to -514.81	-0.09	-0.04 to -0.15
	SFRP4	254.08	100.80 to 407.14	0.07	0.03 to 0.12
	SLITRK1	-352.48	-177.02 to -527.79	-0.10	-0.05 to -0.15
	TCN1	-198.53	-47.63 to -349.31		
	UNC5D	-182.16	-45.71 to -318.51	-0.06	-0.02 to -0.09
**Stratified by girls (n = 163)**	AFM	358.03	157.00 to 558.92		
	CELSR2	-219.83	-84.02 to -354.59		
	EPHA4	-386.26	-229.86 to -542.57	-0.12	-0.07 to -0.18
	SFRP4	232.61	94.68 to 370.54	0.06	0.01 to 0.11
	SLITRK1	-386.99	-239.72 to -534.02	-0.13	-0.08 to -0.18
	TCN1	-207.74	-78.07 to -337.31		
	UNC5D	-232.53	-119.25 to –345.84	-0.08	-0.04 to -0.11
**BWR without adjustment for sex, gestational**	AFM				
	CELSR2				
	EPHA4			-0.10	-0.06 to -0.14

© 2024 Van Pee T et al. *JAMA Network Open.*

**age, and parity (n = 288)**	SFRP4		0.07	0.04 to 0.10
	SLITRK1		-0.11	-0.07 to -0.014
	TCN1			
	UNC5D		-0.06	-0.04 to -0.09

The table shows the difference in the infant's birth weight (gram) and BWR with 95% CI per doubling in cord blood protein levels. Multiple linear regression models were adjusted for sex, gestational age, maternal pre-pregnancy BMI, maternal age at delivery, month and year of delivery, ethnicity, parity, smoking during pregnancy, and maternal education. BWR: birth weight ratio; CI: confidence interval.

© 2024 Van Pee T et al. *JAMA Network Open.*

eTable 6: Sensitivity analyses of associations between cord blood proteins and the infant's body weight or BWR with an interaction term between cord blood protein levels and sex.

Interaction term	Birth weight<br>p-value	BWR<br>p-value
AFM*sex	0.83	
CELSR2*sex	0.97	
EPHA4*sex	0.34	0.25
SFRP4*sex	0.31	0.23
SLITRK1*sex	0.58	0.32
TCN1*sex	0.97	
UNC5D*sex	0.35	0.25

The table shows the p-value of the interaction term on a multiplicative scale between cord blood protein levels and sex in multiple linear regression models between the infant's birth weight (gram) and BWR. Multiple linear regression models were further adjusted for gestational age, maternal pre-pregnancy BMI, maternal age at delivery, year and month of delivery, ethnicity, parity, smoking during pregnancy, and maternal education. BWR: birth weight ratio.

© 2024 Van Pee T et al. *JAMA Network Open.*

eTable 7: Numeric data of the associations between the child's weight, BMI z-score, or waist circumference and cord blood proteins.

Cord blood protein	Weight (kg)			BMI z-score			Waist circumference (cm)		
	Difference	95% CI	p-value	Difference	95% CI	p-value	Difference	95% CI	p-value
AFM	0.30	-0.65 to 1.25	0.53	0.03	-0.31 to 0.36	0.89	0.38	-1.07 to 1.82	0.61
CELSR2	-0.75	-0.08 to -1.42	**0.03**	-0.29	-0.06 to 0.52	**0.02**	-0.90	-1.92 to 0.12	0.08
EPHA4	-1.33	-0.55 to -2.10	**<0.001**	-0.41	-0.06 to -0.52	**0.003**	-1.98	-0.81 to -3.15	**0.001**
SFRP4	0.32	-0.33 to 0.96	0.33	0.08	-0.15 to 0.30	0.49	0.19	-0.79 to 1.16	0.70
SLITRK1	-1.20	-0.48 to -1.92	**0.001**	-0.38	-0.12 to -0.63	**0.004**	-1.62	-0.52 to -2.72	**0.004**
TCN1	-0.03	-0.65 to 0.58	0.92	0.05	-0.16 to 0.27	0.62	-0.04	-0.97 to 0.90	0.93
UNC5D	-0.68	-0.13 to -1.22	**0.02**	-0.23	-0.04 to -0.42	**0.02**	-0.87	-0.04 to -1.71	**0.04**

Table shows the difference with 95% CI for the child's weight, BMI z-score, and waist circumference per doubling in cord blood protein levels. Multiple linear regression models were adjusted for sex, gestational age, maternal pre-pregnancy BMI, maternal age at delivery, date of delivery, ethnicity, parity, smoking during pregnancy, maternal education, and age of the child at the follow-up examination. Statistically significant proteins (p-value <0.05) are indicated in bold. CI: confidence interval.

© 2024 Van Pee T et al. *JAMA Network Open.*

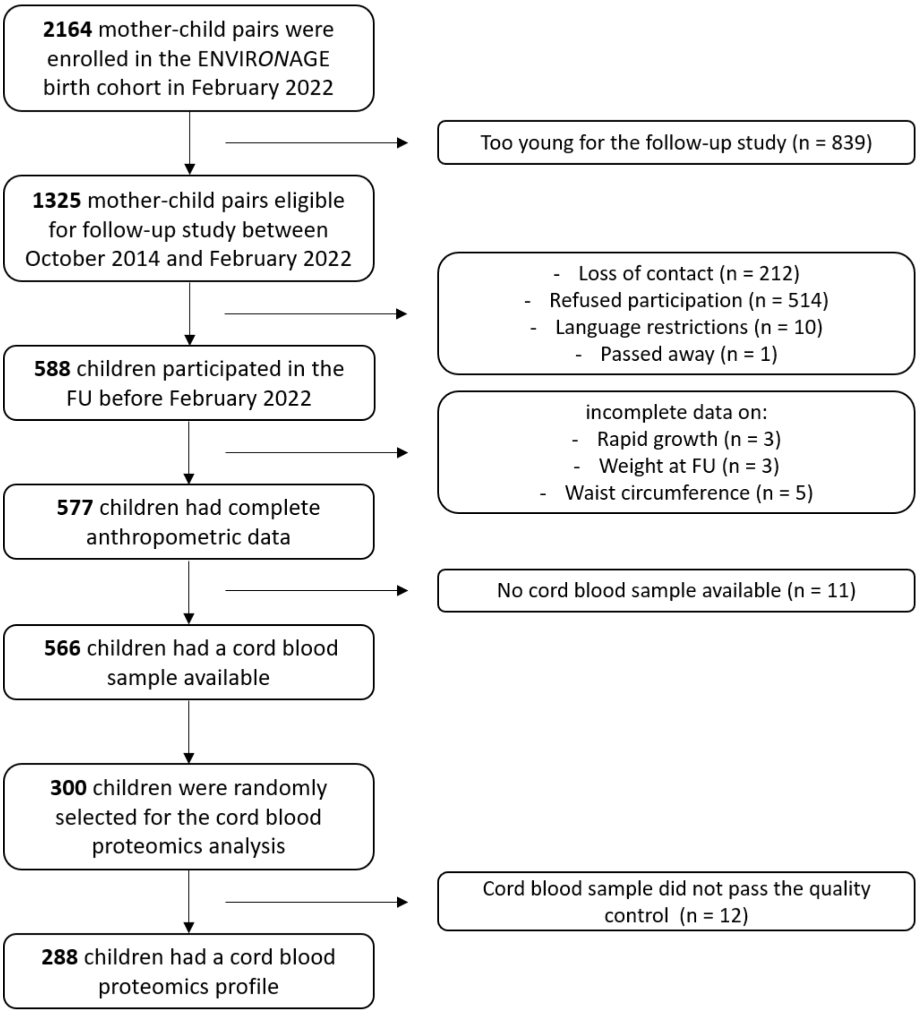

eFigure 1: Participant flow chart depicting the selection of participants enrolled in the ENVIR*ON*AGE birth cohort for arriving at the final study sample size of 288 children. ENVIR*ON*AGE: ENVIRonmental influence ON early AGEing birth cohort; FU: follow-up.

© 2024 Van Pee T et al. *JAMA Network Open.*

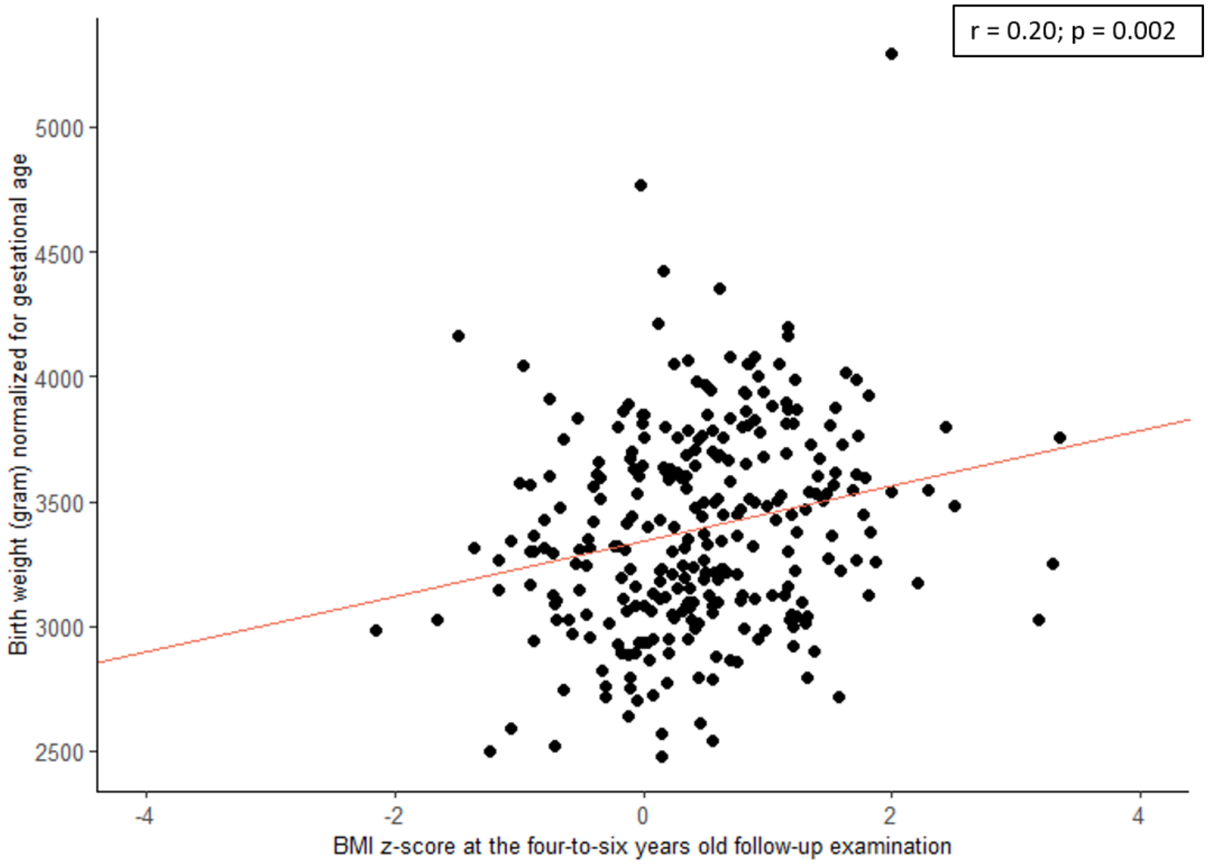

eFigure 2: Scatterplot showing the correlation between the BMI z-score at four-to-six years old and birth weight (grams) normalized for gestational age.

© 2024 Van Pee T et al. *JAMA Network Open.*

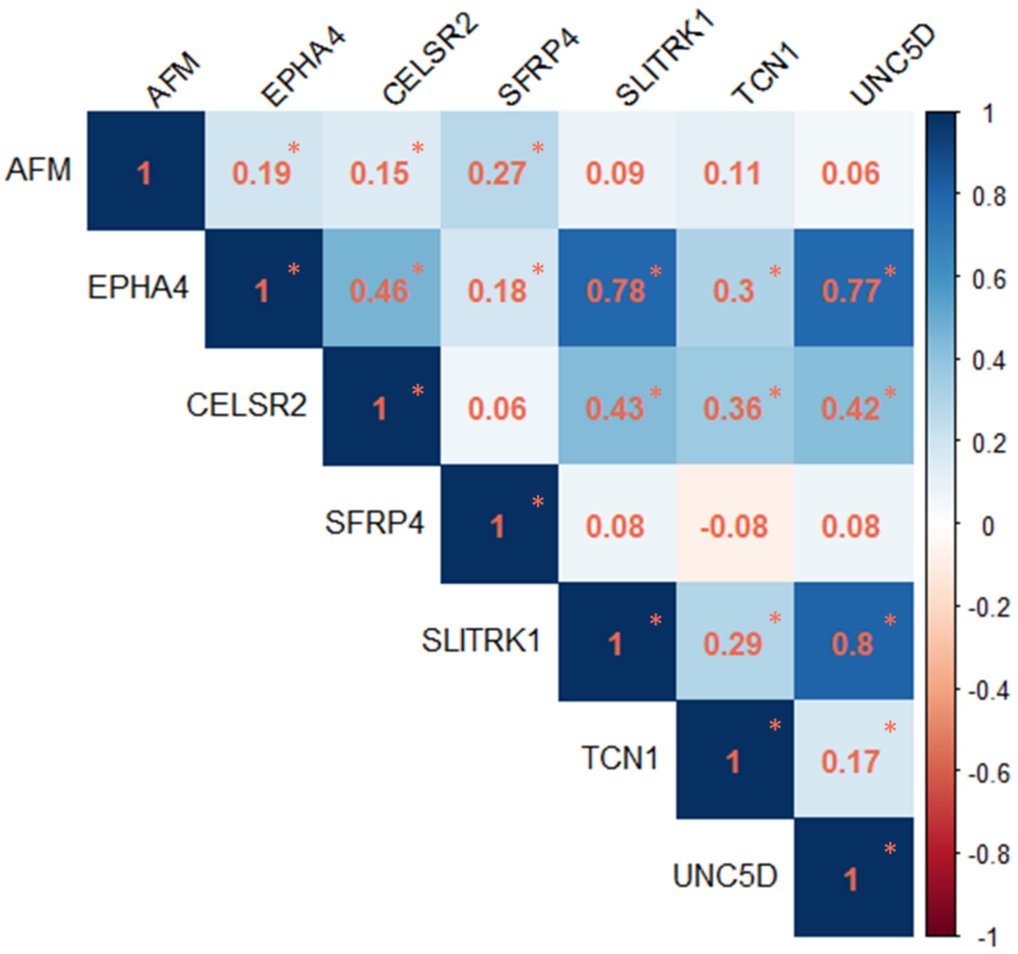

eFigure 3: Pearson correlation matrices between the proteins statistically significant associated with birth weight and/or BWR in the large proteomics analysis. * indicates statistical significance with a two-tailed p-value < 0.05. BWR: body weight ratio.

© 2024 Van Pee T et al. *JAMA Network Open.*

eReferences

1 Armstrong, RA. When to use the Bonferroni correction. *Ophthalmic Physiol Opt* 2014. 34: 502-508 https://doi.org:10.1111/opo.12131

© 2024 Van Pee T et al. *JAMA Network Open.*
