# Supplementary material for: Cord Blood Proteomic Profiles, Birth Weight, and Early Life Growth Trajectories
Source: JAMA Netw Open. 2024 May 14;7(5):e2411246. doi: 10.1001/jamanetworkopen.2024.11246 (PMC11094560; doi:10.1001/jamanetworkopen.2024.11246)
Supplement: Supplement 2. — Data Sharing Statement [file jamanetwopen-e2411246-s002.pdf]

Data Sharing Statement

Van Pee. Cord Blood Proteomic Profiles, Birth Weight, and Early Life Growth Trajectories. *JAMA Netw Open*. Published May 14, 2024. doi:10.1001/jamanetworkopen.2024.11246

Data
Data available: Yes
Data types: Deidentified participant data, Data dictionary
How to access data: Deidentified participant data with an accompanying data dictionary will be made available upon reasonable request to tim.nawrot@uhasselt.be
When available: With publication

Supporting Documents
Document types: None

Additional Information
Who can access the data: Researchers whose proposed use of the data has been approved
Types of analyses: Scientific analyses
Mechanisms of data availability: Signed data access agreement
